# New extracellular factors in glioblastoma multiforme development: neurotensin, growth differentiation factor-15, sphingosine-1-phosphate and cytomegalovirus infection

**DOI:** 10.18632/oncotarget.24102

**Published:** 2018-01-09

**Authors:** Jan Korbecki, Izabela Gutowska, Ireneusz Kojder, Dariusz Jeżewski, Marta Goschorska, Agnieszka Łukomska, Anna Lubkowska, Dariusz Chlubek, Irena Baranowska-Bosiacka

**Affiliations:** ^1^ Department of Biochemistry and Medical Chemistry, Pomeranian Medical University in Szczecin, 70-111 Szczecin, Poland; ^2^ Department of Biochemistry and Molecular Biology, Faculty of Health Sciences, University of Bielsko-Biała, 43-309 Bielsko-Biała, Poland; ^3^ Department of Biochemistry and Human Nutrition, Pomeranian Medical University in Szczecin, 71-460 Szczecin, Poland; ^4^ Department of Applied Neurocognitivistics, Pomeranian Medical University in Szczecin, 71-252 Szczecin, Poland; ^5^ Department of Neurosurgery, Pomeranian Medical University in Szczecin, 71-252 Szczecin, Poland; ^6^ Department of Functional Diagnostics and Physical Medicine, Pomeranian Medical University in Szczecin, 71-210 Szczecin, Poland

**Keywords:** glioblastoma multiforme, cytomegalovirus, neurotensin, growth differentiation factor-15, sphingosine-1-phosphate

## Abstract

Recent years have seen considerable progress in understanding the biochemistry of cancer. For example, more significance is now assigned to the tumor microenvironment, especially with regard to intercellular signaling in the tumor niche which depends on many factors secreted by tumor cells. In addition, great progress has been made in understanding the influence of factors such as neurotensin, growth differentiation factor-15 (GDF-15), sphingosine-1-phosphate (S1P), and infection with cytomegalovirus (CMV) on the ‘hallmarks of cancer’ in glioblastoma multiforme.

Therefore, in the present work we describe the influence of these factors on the proliferation and apoptosis of neoplastic cells, cancer stem cells, angiogenesis, migration and invasion, and cancer immune evasion in a glioblastoma multiforme tumor. In particular, we discuss the effect of neurotensin, GDF-15, S1P (including the drug FTY720), and infection with CMV on tumor-associated macrophages (TAM), microglial cells, neutrophil and regulatory T cells (T_reg_), on the tumor microenvironment. In order to better understand the role of the aforementioned factors in tumoral processes, we outline the latest models of intratumoral heterogeneity in glioblastoma multiforme. Based on the most recent reports, we discuss the problems of multi-drug therapy in treating glioblastoma multiforme.

## INTRODUCTION

One of the most lethal cancers, glioblastoma multiforme (GBM) is the most common cancer of the glial cells, with an incidence of about 3/100,000 persons per year [1–2]. The basic procedure for GBM patients with clinical symptoms caused by the mass effect is surgical treatment (cytoreduction), which is also used to obtain material for histopathological examination. It should be combined with other methods such as radiotherapy, or in another variant chemotherapy with fotemustine or cyclically administered temozolomide (TMZ) or angiogenesis-inhibiting bevacizumab. Other methods, including immunotherapy, continue to be studied [3].

GBM has been subject to highly intense research due to the very low five-year post-operative survival rate, estimated to be only 9.8% [4]. In particular, researchers focus on intercellular signaling in the GBM tumor, i.e. autocrine influence of factors secreted by the GBM cells on themselves and the remaining cells in the niche. This has resulted in significant progress over the last 4 years in the understanding of the previously little known secretory factors such as neurotensin (NT), growth differentiation factor-15 (GDF-15), sphingosine-1-phosphate S1P, and of infection with cytomegalovirus (CMV). In this paper, we begin the discussion of these factors with the issue of intratumoral heterogeneity.

## INTRATUMORAL HETEROGENEITY OF GLIOBLASTOMA

The population of tumor cells is not homogenous. It consists of genetically and epigenetically diverse tumor cells [5–6] with different expressions of mRNA [5, 7] and proteins [8]. This intratumoral heterogeneity in GBM was first reported in the 1980s [9]. Thanks to increasingly precise and sensitive research methods in which proteome, transcriptome and genome analysis can be performed on single cells, recent research shows in detail the differentiation of cancer cells in a GBM tumor [10].

### Formation of intratumoral heterogeneity

Due to the uncontrolled division of cancer cells, a tumor has a much larger number of changed cells at the onset of neoplasm. The divisions result in the accumulation of genetic changes, and over time the environment within the tumor becomes increasingly diverse. In particular, selection pressure is exerted by the distribution of necrotic areas, different concentrations of oxygen including hypoxia [11], metabolic compounds, and tissue hormones, and the placement of unaltered tumor-building cells. Selected in a Darwinian-like manner [10, 12], different tumor cell lines are formed with various mechanisms of bypassing cancer resistance mechanisms, exhibiting properties described as the ‘hallmarks of cancer’ [13, 14].

Intratumoral heterogeneity seems to depend primarily on cancer stem cells, forming a small and rarely dividing population in a tumor [15]. During division they form a stem cell and a rapidly dividing cancer cell. The latter cells have a limited number of divisions and by definition do not form tumors in animals inoculated with them. However, according to most recent research, the differentiated GBM cells are able to dedifferentiate into glioblastoma stem cells (GSC) [16]. This partly refutes the theory of intratumoral heterogeneity based solely on cancer stem cells, and indicates that both stem and differentiated cells are responsible for the diversity of tumor cell lines [16].

### Intratumoral heterogeneity in the development of glioblastoma

Mutations in the development of individual GBM lines are not haphazard. Sottoriva et al. show that they can be organized into three stages [10]. First, very characteristic changes occur on chromosome 7, with the amplification of the fragment with epidermal growth factor receptor (*EGFR)*, cyclin-dependent kinase (*CDK)6*, and *MET* genes. It is also highly likely that deletion occurs on chromosome 10 with the *PTEN* gene. This stage is also characterized by deletion of the chromosome 9 fragment with the cyclin-dependent kinase inhibitor 2A and 2B (*CDKN2A/B*) gene.

The next stages of tumor growth include very different mutations on different chromosomes, which results in a very large diversity of tumor cell lines within a single tumor. These include changes on chromosome 17 with *P53* and neurofibromin 1 (*NF1)* genes, or on chromosome 4 with solute carrier family 2 member 9 (*SLC2A9/GLUT9)* gene, and platelet-derived growth factor (*PDGFR)A* amplification [10]. Also mutations of this type occur later in GBM recurrences, resulting in considerable genetic differences between the GBM cells in the relapse sites and the parent tumor [8].

The probability of each mutation depends on the tumor microenvironment and the selection of individual clones by anti-cancer mechanisms. Of particular significance is the location of the tumor in the brain; e.g. periventricularly located GBM has a higher expression of factors such as vascular endothelial growth factor (VEGF)-C or hepatocyte growth factor (HGF) than at cortical locations [17].

Intratumoral heterogeneity results in the creation of a tumor with a specific cell distribution pattern. GBM cells with amplified *PDGFRA* form a compact population surrounded by cells with amplified *EGFR* [18]. The accumulation of changes results in the formation of specific GBM subtypes: classical, mesenchymal, neural, and proneural [5]. In each GBM tumor there is a proneural cell population [5], while the other subtypes may occur in very low numbers or not at all. However, there have been no studies showing the detailed structures formed by cancer cells.

### Functional domains of the tumor

Experiments on neurospheres derived from stabilized GBM cell lines demonstrate that these tumor cells are interdependent and specialized in specific functions [19]. In particular, tumor cells co-operate with each other for specific purposes in cancer development [20]. An example of this are the mesenchymal GBM cells, which contain many more proteins associated with immunosuppression [21]. Thanks to this they can participate in cancer immune evasion. However, intratumoral functional domains require further research which could open new possibilities for effective antitumor therapies.

### Impact on therapy

GBM cell differentiation in a single tumor in terms of resistance to anti-cancer drugs has very negative consequences for therapy. It is estimated that 1/4 of tumor clones are resistant to TMZ and only 1/10 are very susceptible to the drug [22]. Such a scope of resistance in a GBM tumor is similar for other anti-cancer drugs [22] This has important implications for therapy, because the use of an anti-cancer drug, including TMZ, destroys only those cells which are susceptible to the drug, but leaves other cells that are resistant to it [22]. Within a few months of chemotherapy, new tumors in relapse sites are formed by GBM cells which survive treatment [4]. This results in a five-year survival rate of 10% in patients after chemotherapy with TMZ.

**Figure 1:**
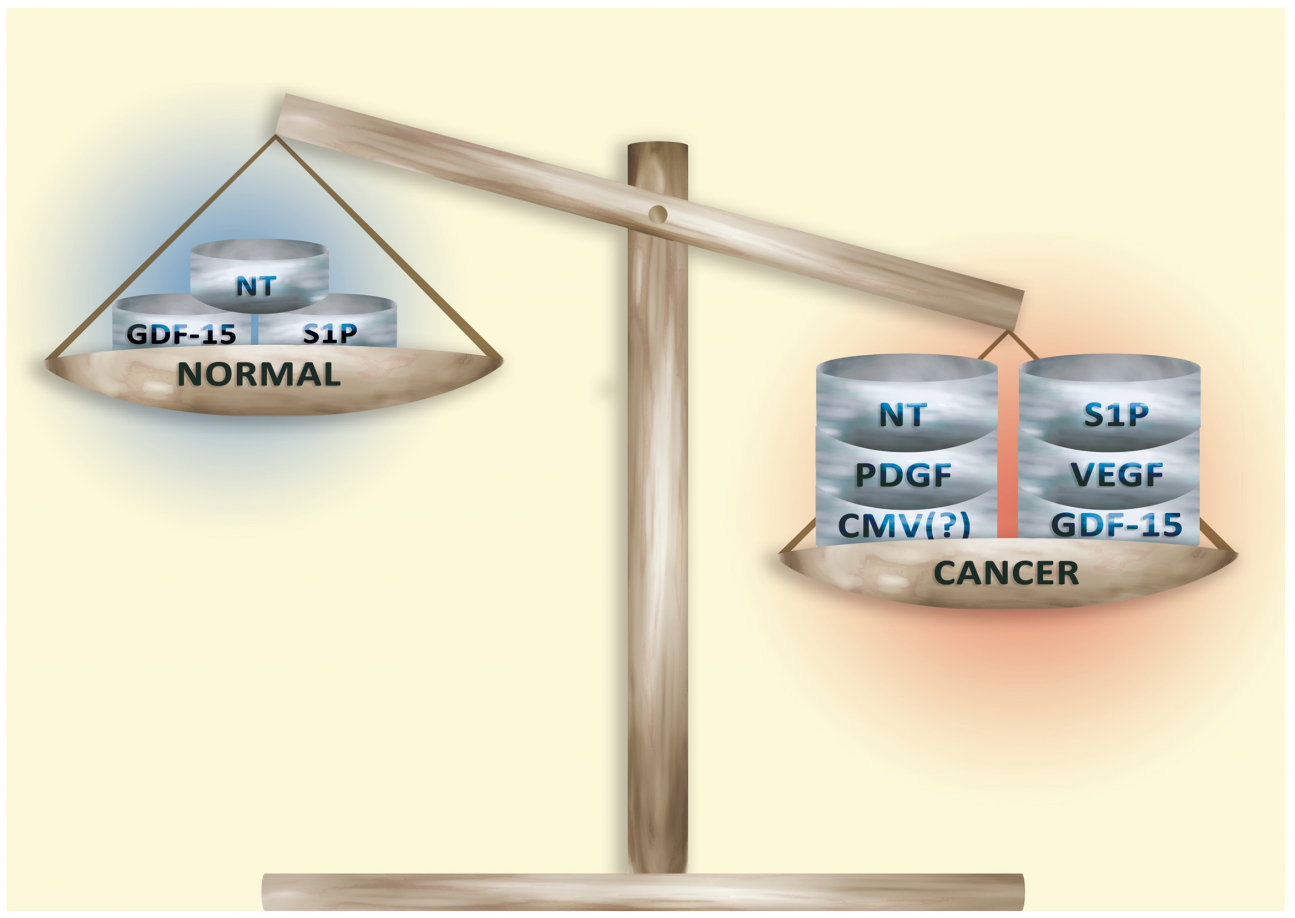
Secretory factors in normal tissue and in the tumor microenvironment Secretory factors responsible for the ‘hallmarks of cancer’ occur in low concentrations in non-cancerous tissue. However, the development of a tumor increases the concentration of these factors. This process is non-specific and so the combinations and levels of secretory factors vary among tumors and even within a single tumor.

Some hope lies in studying the cancer microenvironment, in particular interactions between the tumor niche and cancer cells, and the intercellular signaling in the tumor microenvironment. These processes depend on many secretion factors (Figure 1).

**Figure 2:**
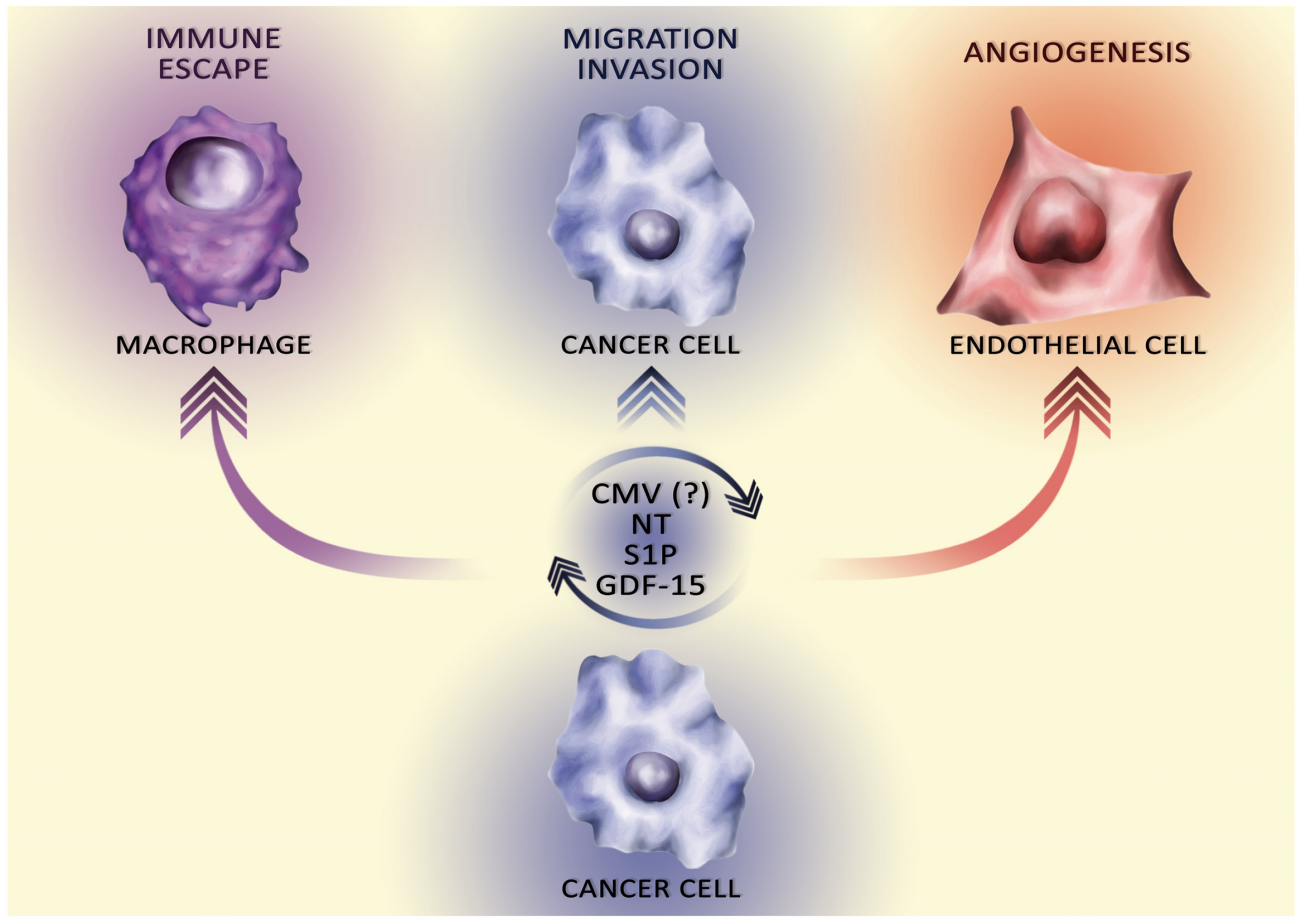
The influence of secretory factors on the ‘hallmarks of cancer’ Cancer cells secrete various secretory factors into the tumor microenvironment. The total pool of these secretory factors affects the hallmarks of cancer, in particular via autocrine stimulation of tumor cell proliferation, angiogenesis, migration and invasion, and tumor immune escape.

GBM has been studied extensively for NT, GDF-15, S1P, and infection with CMV, which play important roles in tumor processes, in particular the viability, migration and invasion of tumor cells, GSC, angiogenesis, and tumor immune escape (Figure 2).

## CYTOMEGALOVIRUS

### Cytomegalovirus as a carcinogenic factor in glioblastoma

CMV is a DNA virus of the β-herpesvirinae subfamily, carried by more than half the global population [23]. Since Cobbs et al. demonstrated the expression of CMV in all GBMs [24], the incidence of CMV infection in the tumor has been widely discussed. Several research groups have confirmed the occurrence of CMV DNA and the expression of antigens of the proteins encoded by its genome in almost 100% GBMs [25–32].

Other research groups have also confirmed the presence of CMV in GBM, although only in 36% (27/75) [33], 51% (25/49) [34] and 75% (12/16) [35] of the tumors studied. In addition, other research groups have shown a lack of CMV infection in samples of brain slices affected by GBM [36–39]. They also postulate a false positive in other research groups due to the cross-reactivity of antibodies with non-viral proteins such as myelin basic protein or human serum albumin [38]. Some also postulate a false positive caused by non-specific immunocytochemistry staining of glial cells with gemistocytic morphology [36].

### Epidemiology of the cytomegalovirus

CMV infection occurs in more than a half of the global population, with the likelihood of infection increasing with age [40–44]. Forty percent of people under 10 years of age are carriers of this virus. Higher age is associated with a higher likelihood of contracting and carrying this virus. In older people, the virus is estimated to have infected 70%-90% of the population, depending on the population studied. Although this high number of CMV carriers is not reflected in the incidence of GBM (3/100,000 persons/year [1, 2]), numerous studies show that the virus does increase the aggressiveness of GBM [45–51].

### Tumor microenvironment and cytomegalovirus

**Figure 3:**
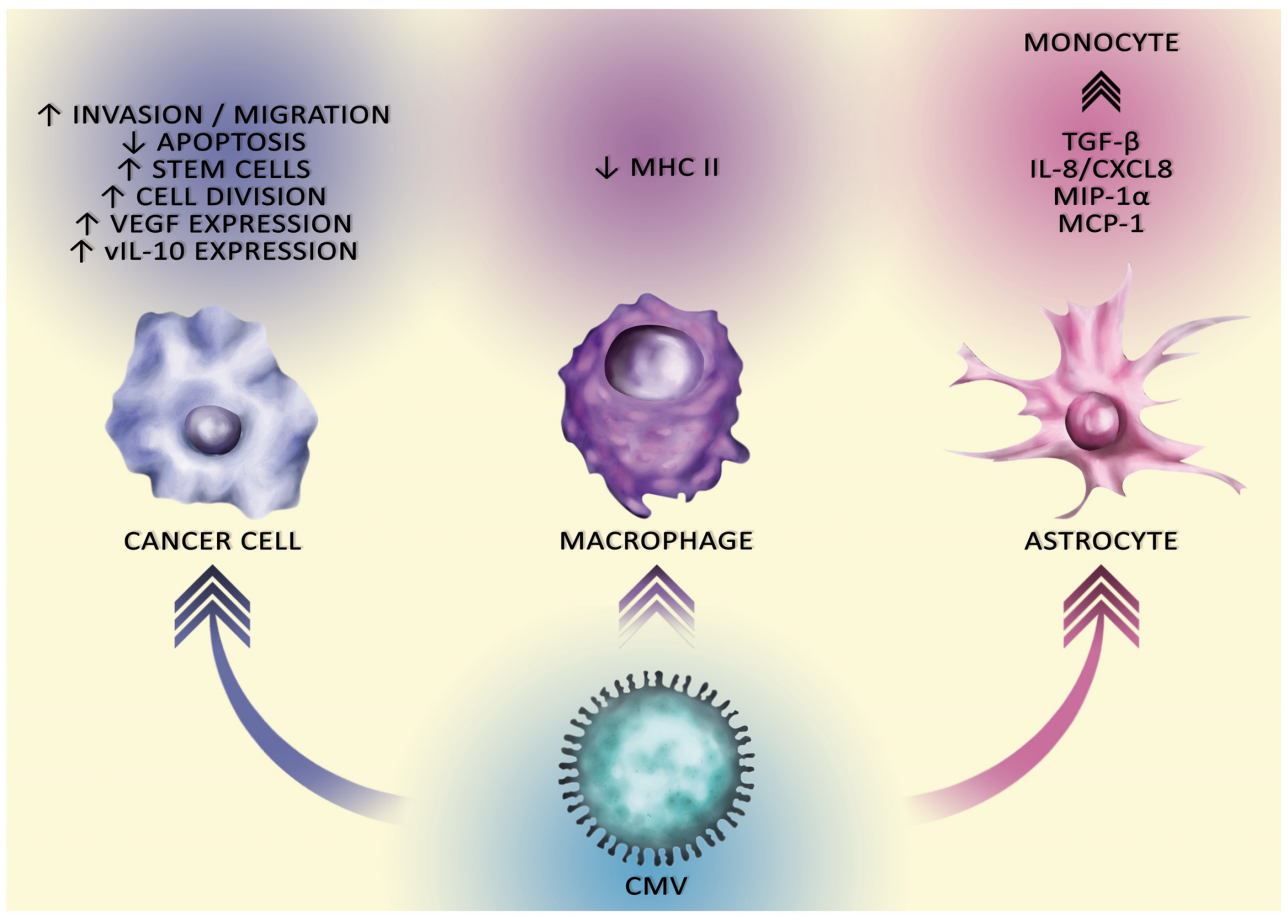
The carcinogenic effect of CMV on different cells CMV affects cells in the tumor niche, particularly macrophages and astrocytes which affects the tumor immune escape. CMV also affects cancer cells. It stimulates migration and invasion GSC, stimulates divisions and disturbs apoptosis. CMV in the tumor cell is responsible for the tumor immune escape via the production of vIL-10.

The appearance of CMV in GBM may be caused by an immunosuppressive microenvironment of the tumor, as CMV infection is completely controlled by a healthy immune system [52–55]. Particularly crucial here is the NK cell response [52, 53, 55]. As a result, the virus exists in the body in a latent form and is reactivated when immunity reduces, e.g. as a result of the action of immunosuppressive drugs after transplantation [56, 57]. The tumor microenvironment, in particular in GBM, also involves intensive immunosuppression processes that cause the immune evasion of cancer cells (Figure 3) [58, 59]. This allows an intensive replication of CMV in GBM [60].

### Effect of cytomegalovirus on the tumor niche and tumor immune escape

Due to the lack of appropriate research models, little is known about the direct influence of CMV on processes occurring in the GBM tumor niche. Nevertheless, *in vitro* experiments with CMV infection of various cell types have made it possible to develop a model of the interaction between this factor and processes occurring in the tumor niche.

### From monocytes with latent infection to lytic infection in the tumor

CMV is present in CD34^+^ bone-marrow progenitors, and consequently it also occurs in peripheral blood mononuclear cells during differentiation of the progenitors into monocytes [61, 62]. CMV does not cause lytic infection of monocytes; it is latent in these cells [63]. However, even during latent infections, the CMV reprograms the expression profile of some of the genes in the monocytes. In this state, only a small number of viral genes [64] are expressed.

Studies have shown increased expression of viral chemokine scavenger receptor US28 that stimulates the migration of infected cells in response to a wide spectrum of chemokines [65, 66]. US28 increases migration especially in response to C-X3-C motif chemokine ligand (CX3CL)1/fractalkine [67] and to a lesser extent in response to chemokines such as CC motif chemokine ligand (CCL)2/monocyte chemoattractant protein 1 (MCP-1), CCL3/macrophage inflammatory protein (MIP)-1α, CCL4/MIP-1β and CCL5/regulated on activation, normal T-cell expression and secretion (RANTES) [65, 67].

The effect of individual chemokines is cell-specific and depends on the type of cell where the expression of US28 has taken place [67]. Also, simultaneous activation of CX3CL1/fractalkine together with CCL2/MCP-1 or CCL5/RANTES on US28 results in no migration of monocytes or macrophages that are expressing this receptor [67, 68]. If in the tumor microenvironment there are also other chemokines (i.e. in addition to CX3CL1/fractalkine), then it results in the inhibition of US28-dependent monocyte and macrophage migration.

CX3CL1/fractalkine is mainly secreted by neurons [69]. In physiological conditions it shows a neuroprotective action, suppressing excessive activation of microglial cells by proinflammatory agents, e.g., LPS or CMV [70, 71]. CX3CL1/fractalkine is also produced by GSC [72] and TAM in the GBM niche [73], which may result in the recruitment of monocytes with latent CMV infection expressing US28. The action of this chemokine may also affect the location of infected TAM and other cells throughout the tumor niche [66, 67].

The use of antibodies neutralizing CX3CL1/fractalkine results in a decrease in the intensity of the migration of TAM and microglia isolated from GBM tumors. Nevertheless, the use of CX3CR1-neutralizing antibodies, which are the specific receptor for this chemokine, causes the same decrease in the intensity of migration of these cells [74]. This demonstrates that in the tumor microenvironment in these cells, it is CX3CL1/fractalkine with CX3CR1, but not viral US28, that are responsible for the migration of TAM and microglial cells. The effect of CX3CL1/fractalkine-dependent TAM and microglial cells in the context of CMV infection requires further research, especially with regard to the recruitment of monocytes with latent CMV infection.

After the migration into the tumor niche, monocytes differentiate into macrophages [75]. This differentiation of monocytes with latent infection into macrophages often causes CMV reactivation [61, 62]. CMV reactivation can also be caused by granulocyte-colony stimulating factor (G-CSF) [76], a cytokine produced in the GBM tumor [77]. These facts may explain the presence of active CMV infection in GBM.

### Cytomegalovirus as a oncogenic factor: effect on apoptosis and proliferation

**Figure 4:**
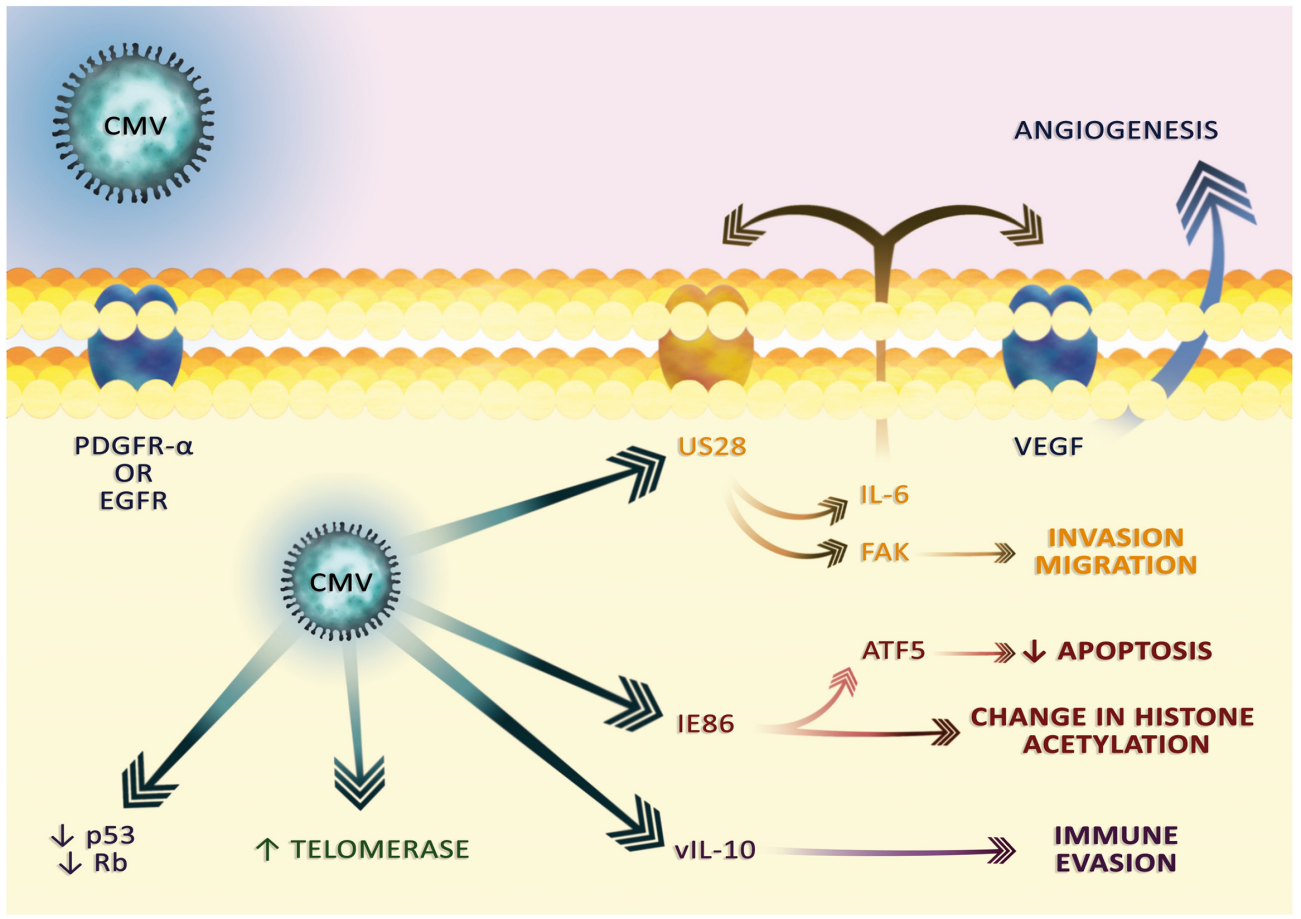
The cytoplasmic effect of CMV infection CMV virions enter cells via the PDGFR-α or EGFR receptors. The viral proteins transmit the signal that causes changes characteristic for cancer. In particular, the US28 viral receptor is involved in angiogenesis, migration and invasion. vIL-10 is involved in tumor immune escape.

Some researchers suggest that CMV occurs in almost all GBMs and the incidence of CMV in GBM tumors is positively correlated with the grade of the tumor. Indeed, almost all GBM samples with the highest grades have been reported to contain the antigens or DNA of this virus [25–32]. This indicates that CMV plays an important role in the development of GBM. As demonstrated by *in vitro* experiments, the virus enters GBM cells through EGFR [78] or PDGFR-α (Figure 4) [79]. These receptors are important for GBM cells and are often amplified and overexpressed [80, 81]. CMV has a particular tropism to GSC in which it enhances the stem cell phenotype [46, 48–50]. CMV in tumor cells disrupts apoptosis in many ways, especially via the viral proteins, such as activation of the viral inhibitor caspase-8 and the viral mitochondria-localized inhibitor of apoptosis, homolog of anti-apoptotic Bcl-2 [82]. In addition, the immediate early 86 (IE86) viral protein initiates activating transcription factor 5 (ATF5), an anti-apoptotic protein commonly found in GBM [31, 83]. The IE86 protein also causes changes in the level of histone acetylation, which changes the expression of many genes in GBM cells [31]. In addition to its effect on apoptosis, CMV also affects cell division. It enhances the expression of telomerase, an enzyme essential for unlimited cancer cell divisions [84]. Viral proteins also reduce the expression of Rb and p53 proteins, which are important for regulating cell division [45, 85–87]. In addition, CMV proteins alter the expression of cell cycle cyclins, halting the division of normal cells and favoring viral DNA replication [88, 89]. However, as a result of tumor changes, this mechanism is impaired and CMV in some GBM cell lines induces cell division [45]. Another mechanism in which viral proteins promote GBM growth is the activation of the PDGFR-α and phosphatidylinositol-4,5-bisphosphate 3-kinase (PI3K)-protein kinase B (PKB) pathways, i.e., pathways crucial for the stimulation of GBM proliferation [45, 90, 91].

### Effect on angiogenesis and tumor dissemination

In addition to the effect on replication, CMV increases angiogenesis and GBM dissemination. The CMV genome encodes the US28 receptor, a homolog of the receptor for the CC chemokine family, responsible for the disruption of the immune response against CMV [23]. US28 increases the expression of VEGF by increasing interleukin (IL)-6 expression and activating the hypoxia inducible factor (HIF)-1α/pyruvate kinase M2 (PKM2) pathway [51, 92–94]. IL-6 activates its receptor and signal transducer and activator of transcription (STAT)3, which then expresses VEGF. In addition to the effects on VEGF, CMV also reduces the expression of thrombospondin-1, an angiogenesis inhibitor [87]. CMV infection of the GBM cells also results in increased expression of endocan, a compound associated with the remodeling of the blood vessels and angiogenesis [95].

CMV also participates in a very characteristic sign of GBM, i.e., early cancer dissemination. US28 activates focal adhesion kinase (FAK) via phospholipase C-β (PLC-β), an enzyme reducing the adhesion of cells, which results in the migration of GBM [93, 94, 96, 97]. Another way of dissemination is in increased expression of matrix metalloproteinase (MMP)2 [35]. However, the mechanisms of CMV's influence on GBM cells requires further research and more detailed understanding, in particular regarding the activation of the human endogenous retrovirus [98].

Another mechanism of CMV-induced angiogenesis in GBM is intensification of the stem cell phenotype [48]. Finally, the CMV genome contains a UL7 protein, similar to the N-terminal V-like domain of carcinoembryonic antigen-related cell adhesion molecule 1 (CEACAM1), inducing vasculogenesis and migration of endothelial cells [99].

### Effect on cancer cells in cancer immune evasion

CMV affects the communication between the tumor cell and the cell of the tumor microenvironment. In particular, the virus causes immune evasion of the infected cancer cell, especially GSC [46]. It disrupts major histocompatibility complex (MHC) I: human leukocyte antigen (HLA)-A, HLA-B and HLA-C, expression of which makes it impossible to recognize the altered antigens on the tumor cell by immune cells [100–104].

In infected cells CMV also reduces the expression of surface HLA-G and increases the levels of soluble HLA-G, as evidenced by experiments on U-373 MG astrocytoma cells [105, 106]. HLA-G is a non-classical molecule of MHC I that plays an important role in maternal-fetal tolerance [107] but is also a carcinogenic factor [108]. Lowering surface HLA-G expression and increasing the expression of soluble HLA-G by CMV is a mechanism of viral attack on the host immune response.

There are cytotoxic T cells in the human body that recognize CMV antigens response restricted by HLA-G [109]. However, in the tumor microenvironment, the increased amount of soluble HLA-G has an immunosuppressive effect [108, 110]. Although the degradation of surface HLA-G may stimulate an antitumor immune response, at the same time numerous cancer immune evasion mechanisms occur in the tumor microenvironment.

CMV infection of GBM cells also results in increased expression of arginase-2 [111] and FasL [112], which interfere with cancer-related immunosuppression. In an infected cell, CMV also induces the production of viral interleukin-10 (vIL-10) with an immunosuppressive action [47, 113, 114]. Infected cells also produce various chemokines that cause chemotaxis of various cells to the site of the CMV infection. If a CMV infection focus is present in the GBM tumor, then these infected cells maybe be recruited into the tumor niche.

### Effect of cytomegalovirus on astrocytes

CMV has a high tropism for astrocytes and therefore these cells play an important role in CMV infection of the brain [115]. CMV has tropism for neural stem cells and immature glia cells in the subventricular zone [116, 117], and for GSC in the tumor niche [46]. CMV is also replicated in other cells, including nerve cells [118]. Infection in the brain is followed by chronic inflammation in this organ. Viral processes and the fight against CMV crucially depend on the production of chemokines, i.e. cytokines with chemotactic activity.

In the first stage of infection, astrocytes increase the expression of chemokines such as CCL2/MCP-1, CCL3/MIP-1α and IL-8/CXC motif chemokine ligand (CXCL)8, but not CCL5/RANTES. However, the infected astrocytes do not produce an increased amount of proinflammatory cytokines such as IL-1β, IL-6 or tumor necrosis factor α (TNF-α) [119]. Chemokines enable the chemotaxis of immune cells responsible for the fight against CMV infection. In combination with pro-inflammatory cytokines [120, 121] produced by other immune system cells such as NK cells, NKT cells, microglial cells [89, 122], CD4^+^ T-cells [123] and CD8^+^ T-cells [124], the fight against CMV infection can proceed. CMV also causes a decrease in the expression of CCR5, which interferes with the chemotaxis of these cells in response to chemokines [125]. This effect is dependent on viral protein UL128, which is included in the envelope of the CMV virion [126]. During cell infection, this protein degrades several chemokine receptors, not just CCR5, to interfere with chemo-dependent migration of infected cells.

Chemokines which play an important role in fighting CMV infection in an immunosuppressive cancer microenvironment can support tumor processes. Secreted by cells infected with CMV, chemokines act as chemoattractants for regulatory T cells (T_reg_), microglia, neutrophils and monocytes. These cells are recruited into the GBM tumor niche where they participate in tumor processes [59, 127–129]. CMV infection results in increased expression and secretion of cytokines that may contribute to the formation of the immunosuppressive cancer microenvironment. In particular, expression of vIL-10 in astrocytes plays a crucial role in immunosuppressive mechanisms [122, 130]. This cytokine reduces the production of CXCL10/IP-10 chemokine in infected microglial cells and thus reduces recruitment of lymphocytes that fight against CMV and tumor cells [122]. vIL-10 also causes differentiation of monocytes into immunosuppressive macrophages with a M2c phenotype [47, 130]. In the infected astrocytes transforming growth factor β (TGF-β) is expressed, an immunosuppressive cytokine having a key function in the development of GBM [131].

In addition to the effects on immune responses, CMV in astrocytes disrupts the uptake of glutamate [132]. This results in an increase in the concentration of this amino acid and cell toxicity in the CMV infection microenvironment [132]. This feature is shared with GBM [133]. However, there are no studies on CMV dependence on cytotoxic concentrations of glutamate in the GBM microenvironment.

### Effect of cytomegalovirus on microglial cells

CMV infection causes major changes in microglial function. CCL2/MCP-1 and CCL5/RANTES chemokines produced in infected astrocytes cause migration of microglial cells to the site of CMV infection [119, 134, 135]. Influenced by CMV, microglia produce pro-inflammatory cytokines such as IL-1β, IL-6, and TNF-α, which help to control CMV infection. Infected microglial cells also produce chemokines such as CCL2/MCP-1, CCL3/MIP-1α, CCL5/RANTES, IL-8/CXCL8 [119] and CXCL10/IP-10 [122]. CMV infection also results in the degradation of CCR5 and CXCR4 in microglia [125], as well as other chemokine receptors, which interferes with chemokine-dependent chemotaxis of infected cells [126]. This process depends on the aforementioned CMV virion envelope protein: UL128.

CCL2/MCP-1 and CCL5/RANTES cause monocyte migration, which results in the accumulation of these cells in the site of CMV infection [136]. In turn, CXCL10/IP-10 induces migration of T and NK cells [122], which results in the accumulation of CD8^+^ T-cells, responsible for chronic activation of microglial cells via the production of interferon gamma (IFN)-γ [124] which helps to control CMV infection. However, chronic inflammation is toxic to the cells in the brain, and one of the mechanisms that protects the brain from damage are immunosuppressive reactions involving T_reg_ [124].

Chronic inflammation can assist in the development of many cancers, including GBM. Prolonged inflammation activates immunosuppressive mechanisms. In particular, the effect of IFN-γ changes to oncogenic [137]. Chronic inflammation is also accompanied by the recruitment of T_reg_ [138] and an increase in the expression of immunosuppressive IL-10 [139]. In addition to the inflammatory effect, infected cells produce chemokines: CCL2/MCP-1 and CCL5/RANTES and also chemokines encoded by the CMV genome, that help in recruiting various GBM-associated cells, including neutrophils [139–144], macrophages [135, 136, 145, 146], microglial cells [119, 134, 135] and T_reg_ [129]. After migration, these cells are included in the carcinogenic mechanisms.

In particular, this refers to the presence of anti-inflammatory cytokines in GBM, such as IL-10 and TGF-β, and also vIL-10 expression during CMV infection. The latter cytokine reduces expression of CXCL10/IP-10 in microglial cells and thus reduces recruitment of lymphocytes fighting against CMV infection and responsible for tumor destruction [122]. Also, vIL-10 causes monocyte differentiation into immunosuppressive macrophages with M2c phenotype [47, 130]. The anti-inflammatory cytokines, in particular vIL-10, are responsible for the expression of programmed death ligand-1 (PD-L1)/B7-H1 in microglia [46, 147]. This is an immunosuppressive molecule that reduces the antiviral response of CD8^+^ T-cells but also the antiviral activity of other immune cells. This mechanism also contributes to GBM tumor immune evasion. However, due to the lack of appropriate research models, this requires confirmation, just as the other CMV activities in GBM.

### Influence of infection in tumor niche on monocytes and macrophages

In addition to CX3CL1/fractalkine, other chemokines also affect monocyte chemotaxis into the GBM tumor. In particular, CMV-infected GSC cells [46], astrocytes [119], macrophages [63, 148, 149] and microglia [119] secrete increased amounts of CCL2/MCP-1 and CCL5/RANTES. It seems that expression of these chemokines, at least with regard to CCL2/MCP-1, is highest in the early phase of cell infection and is dependent on the pp71 viral protein [63, 150, 151]. The expression of some chemokines is then reduced by other viral proteins at a later stage of infection [63, 150, 151].

CCL2/MCP-1 and CCL5/RANTES are chemokines that cause chemotaxis of monocytes from the blood and the subsequent accumulation of these cells in the focus of CMV infection. These chemokines also play an important role in the recruitment of monocytes into the cancer niche [135, 136, 145, 146]. Also, the CMV genome encodes viral chemokines that affect monocytes and macrophages. In particular, the murine CMV genome encodes murine cytomegalovirus chemokine (MCK)-1 and MCK-2 [152, 153]. This shows the identical mechanisms of CMV and GBM on blood monocytes. However, further studies are required to understand the effects of the aforementioned chemokines secreted during CMV infection on the recruitment of monocytes into the GBM niche.

After chemotaxis of monocytes into the tumor niche, they differentiate into macrophages. This process is induced by IL-10 and vIL-10, encoded by the CMV genome. These cytokines, in particular vIL-10, induce differentiation of monocytes into macrophages with phenotype M2c [46, 47, 113, 114]. Differentiation of monocytes by vIL-10 results in the activation of the PI3K and STAT3 pathways resulting in the increased expression of heme oxygenase-1 (HO-1) [47, 130]. Expression of this enzyme maintains this state of macrophage polarization.

These cells exhibit increased expression of IL-10 [130] TGF-β [46] and VEGF [46] and increased expression of immunosuppressive protein PD-L1/B7-H1 [46]. This is also accompanied by reduced expression of TNF-α [154] and a reduction in the expression of MHC II components [46, 101, 102, 155, 156]. As a result, the impaired MHC II presentation of antigens interferes with the antiviral response and also disrupts the antitumor response of the immune system [104].

One should not forget that CMV infection is not the only factor causing monocyte differentiation into macrophages. In GBM there are other factors that are produced by GBM cells and cells that accompany this tumor. In particular, these are factors such as M-CSF [157] or IL-10 [158]. However, further research is required to understand this problem.

### Influence of direct infection on monocytes and macrophages

Direct infection with CMV affects monocytes [159]. CMV does not replicate in infected monocytes, but these cells are subject to latent infection with this virus [63]. After the infection of monocytes, CMV inhibits the apoptosis of these short-lived cells [160, 161]. This virus disrupts the expression of antigens by the infected monocytes; in particular it lowers the expression of MHC II components, in particular HLA-DR [62, 162].

During direct infection with this virus, monocytes dependent on NF-κB and PI3K differentiate into macrophages that simultaneously secrete cytokines and chemokines of M1 and M2 macrophages [148, 149, 163]. The gene expression profile is more similar to M1 than to M2 polarization [148, 159]. There is an increase in the expression of cytokines associated with M1 polarization, i.e. IL-1β, IL-6, IL-15, TNF-α, and an increase in the expression of M1 marker: CD80. However, infected monocytes also secrete factors associated with M2 polarity, such as IL-10 [148].

Infected monocytes begin to produce larger amounts of chemokines, in particular CCL2/MCP-1, CCL3/MIP-1α, CCL4/MIP-1β and CCL5/RANTES but also large amounts of CCL8/MCP-2, CCL19/ELC, CCL20/MIP-3α, CCL23/MPIF-1, with a reduced secretion of CXCL1/GROα [63, 148]. They also secrete CXCL10/IP-10 and CXCL11/I-TAC, causing T-cell and NK cell migration with a possible antiviral effect. Expression of CCL2/MCP-1, CCL4/MIP-1β and CCL8/MCP-2 is highest at the onset of monocyte infection and decreases with time [63, 150, 151]. CMV also causes an increase in cyclooxygenase-2 (COX-2) expression in infected monocytes, but also a decrease in VEGF expression [148].

Infection of monocytes with CMV virions results in reduced expression of many receptors for chemokines such as CCR1, CCR2, CCR5 and CXCR4 [148, 164], which interferes with the action of chemokines directly after CMV infection. CMV does not affect the expression of CCR7 and CX3CR, which is already low in monocytes [164]. This effect is dependent on viral protein UL128, which is included in the CMV envelope complexes [126]. During infection, this protein causes the degradation of many chemokine receptors, which may be very important in the GBM tumor, where CMV is intensely replicated.

CMV infects the already polarized macrophages M1 and M2, with a higher tropism for M2 macrophages [149, 165]. After macrophage infection, CMV inhibits apoptosis in these cells [166]. In infected macrophages CMV further increases the expression of surface and soluble HLA-G, which impairs the immune response in the microenvironment of the infected cells. [105]. This may explain HLA-G expression in TAM and microglial cells in GBM tumor sections [167].

Soluble HLA-G also causes monocyte differentiation to immunosuppressive M2 macrophages, which may be significant in a tumor microenvironment with active CMV infection [108, 110]. The infection of macrophages is pro-inflammatory, which stimulates the immune system and thus may have an antitumor effect. The increased expression of MHC I components (HLA-A, HLA-B and HLA-C) and CD80 and CD86 helps in the presentation of antigens by these cells. However, it appears that the effect of CMV on the amount of MHC I is cell-specific because in the U-373 MG astrocytoma cells [105, 106] or primary murine fibroblasts [168], CMV causes MHC I degradation. In M2 macrophages, CMV reduces the expression of their markers: CD163 and CD206 [149].

Cytokine expression also changes in infected macrophages. In M1 there is an increase in the expression of chemokines and pro-inflammatory cytokines [149]. The same effect is exerted by CMV infection of M2 macrophages. There is an increase in the expression of chemokines such as CCL2/MCP-1, CCL3/MIP-1α, CCL4/MIP-1β, CCL5/RANTES but not CXCL10/IP-10 [149]. This helps in recruiting monocytes from the blood to the CMV infection focus, but this mechanism is also common in cancer, not just GBM [136].

Infection of M2 macrophages results in increased secretion of pro-inflammatory cytokines such as IL-1β, IL-2, IL-6, IL-12, IL-15, TNF-α and IFN-γ and anti-inflammatory IL-10. Infected M2 macrophages secrete larger amounts of VEGF, which affects angiogenesis. It is also worth noting that the chemokines secreted by CMV-infected cells affect angiogenesis [169–171]. In particular, CCL2/MCP-1 and CCL5/RANTES cause vascular remodeling which may affect angiogenesis in GBM. Factors secreted by infected M2 macrophages are capable of enhancing immune responses in immune cells, which may have antiviral and antitumor effects.

In addition to the effects on the secretion of cytokines and chemokines, CMV interferes with chemotaxis in infected macrophages. In particular, it reduces the expression of CCR1 and CCR5 [172]. This effect is dependent on the expression of CMV genes. CMV replication also results in the expression and secretion of macrophage migration inhibitory factor (MIF) [172]. In this way, macrophages (also uninfected macrophages) are insensitive to many chemokines such as CCL2/MCP-1, CCL5/RANTES, CX3CL1/fractalkine, as well as to CCL19/MIP-3, CXCL1/GROα, CXCL12/SDF-IL-8/CXCL8 and macrophage-colony stimulating factor (M-CSF) [172]. Nevertheless, CX3CL1/fractalkine causes an *in vitro* increase in the migration of TAM and microglial cells isolated from GBM tumors [74].

The results of studies on the expression profile of different genes in TAM and microglia from a GBM tumor partially coincide with *in vitro* studies on the infection of macrophages. In particular, TAM and microglia isolated from GBM exhibit the expression of proinflammatory cytokines such as IL-1β, IL-6, and TNF-α at a level similar to M1 macrophages [73]. Similar observations in TAM from *in vivo* models in mice show a mixed gene expression profile. In these models TAM simultaneously express genes specific for different macrophage phenotypes, with a predominance of M1 phenotype [173, 174]. However, TAM isolated from postoperative human GBM tumors do not express genes associated with immune activation [175].

TAM from GBM postoperative tumors are significantly different from CMV-infected and non-infected M2 macrophages. In particular, these TAM do not express TNF-α, although 20% of microglial cells and myeloid-derived suppressor cells (DMSC) isolated from GBM tumors do express this cytokine [176]. *In vitro* macrophage infection by CMV increases TNF-α expression [149] which indicates that CMV infection, if present in GBM tumors, does not affect TNF-α expression in TAM. But microglial cells infected *in vitro* by CMV do exhibit enhanced TNF-α expression, indicating the influence of CMV infection [119]. TAM have reduced expression of CD163 and CD206. In particular, these markers are expressed by a very small percentage of TAM isolated from proneural and neural GBM [176]. This is similar to the *in vitro* observation of CMV-infected M2 monocytes, in which CD163 and CD206 expression was reduced [149].

The effect of CMV monocyte infection on the differentiation of these cells in the GBM tumor, as well as the effect on infected TAM, still needs to be investigated further. Nevertheless, some of the findings on TAM isolated from postoperative GBM tumors were in contrast to those expected from *in vitro* studies on CMV-infected macrophages. On the other hand, studies on microglia and DMSC [176] have shown that inflammation caused by some factors match CMV infection. Research on CMV infection in GBM should be continued, with particular regard to the location of the infection in a particular type of cell in the tumor niche.

CMV has different tropisms for different cells. Also, the replication rate of this virus varies between cell types [177]. CMV lytic infection is destructive to the cells to which the virus has a particularly high tropism and a high rate of replication. However, in some cells, the virus immediately goes into a latent state and is activated only by some undiscovered factors. This results in a certain intratumoral heterogeneity in the CMV infection focus.

### Effect of cytomegalovirus on regulatory T cells

Acute CMV infection results in inflammatory reactions and, in particular, chronic activation of microglial cells [124]. Immunosuppressive reactions, in particular recruitment of T_reg_, help to reduce excessive inflammatory response and thus protect against brain damage [138]. During cessation of the inflammatory response, the concentration of T_reg_ in the inflammatory focus returns to physiological levels.

In a GBM tumor there is an increased number of T_reg_ that have a role in cancer immune evasion [178]. Recruitment of these cells is accomplished via CCL2/MCP-1 [129], i.e. a chemokine that is produced by CMV-infected cells [89]. Further research is required to determine whether CMV affects the recruitment of T_reg_ into the tumor niche or the expression of CCL2/MCP-1 is the result of CMV-independent cancer mechanisms. Further studies are also needed with regard to T_reg_ populations in GBM tumors and how they are influenced by CMV. CMV carriers, particularly older adults, have increased numbers of cytomegalovirus-induced regulatory T cells (iT_reg_) [179–181]. These are T_reg_ which alleviate inflammatory reactions. However, iT_reg_ are specifically activated by CMV antigens, which causes them to only act in the focus of the CMV infection.

CMV has been shown to activate a certain T-cell subpopulation to produce IL-10 and thereby to alleviate the immune response. These cells do not express Foxp3, a T_reg_ marker [182]. This T-cell subpopulation is activated in response to IL-27, which in turn is induced by type I IFN. These chemokines are produced by infected cells. In particular, IFN-α is produced in infected monocytes [148, 183] and IFN-β in infected M2 macrophages [165]. Further studies on the effect of CMV on T_reg_ and on other immune system cells in a GBM tumor are required.

### Effect of cytomegalovirus on neutrophils

Neutrophils play an important role in reactions caused by CMV which infects these cells and thus is spread throughout the body [184]. They also play an important role in GBM. Neutrophils are recruited near CD133^+^ GSC [185], i.e., near the same cells for which CMV has tropism [46]. The elevated number of neutrophils in the GBM tumor increases the aggressiveness of this tumor and, in addition, worsens the prognosis for the patient [186, 187]. Neutrophils in tumors are involved in angiogenesis, migration and invasion of cancer cells, and cancer immune evasion [127]. However, very little research has been devoted to the relation between these cells and cancer.

Neutrophils have been shown to be recruited under the influence of chemokines which are expressed in CMV-infected cells. The chemokines that are important for neutrophils include IL-8/CXCL8 [140], CCL2/MCP-1 [139] and viral CXC motif chemokine ligand 1 (vCXCL1) [141–144]. The CMV genome contains the UL146 gene which encodes protein vCXCL1. This viral chemokine, which works specifically as a chemoattractant for neutrophils, allows CMV to infect neutrophils and spread throughout the body in these cells [184]. If CMV infection is present in the GBM tumor, then neutrophils may be recruited into the tumor niche. However, the exact effect of CMV on neutrophil recruitment, as well as the effect of this carcinogenic factor on already recruited neutrophils in GBM is poorly understood and requires further investigation [186].

### Correlation between cytomegalovirus infection and glioblastoma epidemiology

All of the discussed mechanisms may play a crucial role in GBM growth, which may be confirmed by the fact that some research groups estimate that 100% of GBM are infected with CMV [25–30, 32]. This virus also very often causes congenital neuronal disorders. It is striking that CMV has tropism for neural stem cells and immature glia cells in the subventricular zone [116, 117]. This region of the brain is considered to be the source of stem cells from which cancerous tumors such as gliomas (including GBM) are produced via carcinogenesis [188]. However, over 50% of the population has a latent CMV infection [40–43] and the number of GBM cases is only about 3/100,000 persons per year [1, 2], which shows a poor correlation between CMV infection and GBM epidemiology. CMV infection models in GBM in mice should answer further questions about the exact role of CMV in GBM development [189].

## NEUROTENSIN

### Neurotensin, receptors, functions

Neurotensin (NT) is a peptide hormone consisting of 13 amino acids. There are currently 4 known receptors of this hormone: NT receptor types 1-4 (NTSR_1-4_) [190]. NTSR_1_ has a high (0.1-0.3nM) affinity for NT, and NTSR_2_ has a low (3-10nM) affinity. Both these receptors are G-protein-coupled. Two other receptors, NTSR_3_/sortilin and NTSR_4_/SorLA, contain the Vps10p domain [191]. The extracellular domain of the NTSR_3_/sortilin can be released by its proteinase, and as a result can occur as soluble NT receptor type 3 (sNTSR_3_), performing biological functions without the involvement of NT [192, 193].

NT regulates the function of the digestive tract [194, 195]. In particular it stimulates the small bowel as well as colonic mucosa growth, and increases the production of digestive enzymes by the pancreas. NT is also produced in the brain where it influences the secretion and action of neurotransmitters [196–198]. In particular, NT reduces the effect of dopamine [197, 198]. NT also causes an increase in extracellular glutamate levels, associated with neurotoxic effects in pathological conditions [199–201]. NT is therefore associated with neurodegenerative diseases, in particular Parkinson's disease as well as schizophrenia or drug abuse [197]. Finally, NT is also associated with cancer, which has been best researched in pancreatic, colorectal, breast, lung, prostate, and liver cancers [194]. Recent research shows that NT has important functions in gliomas, especially in GBM [202].

### Neurotensin and cancer cell

Expression of NT and NTSR_1_ in gliomas increases with increasing tumor grade [202]. Among the gliomas, the highest expression of NT and NTSR_1_ occurs in GBM, which positively correlates with increased postoperative mortality [202]. In addition, different cell lines express different NT receptors. The GL261, U-87 MG, U-118 MG and A172 lines express NTSR_1_ [202–204]. The C6 line does not express NTSR_1_ but rather NTSR_2_ [205]. The U-373 MG line expresses three different NT receptors: NTSR_1_, NTSR_2_ and NTSR_3_/sortilin [206].

### Effect on signal transduction in tumor cell

Exact NT signal transduction in GBM cells is not well known. Exact mechanisms have been established in other cancers, mainly lung, breast, colon and pancreatic adenocarcinoma cell lines [192, 207–210]. Activation of the NTSR_1_ receptor leads to activation of the EGFR family: in particular EGFR, ErbB2/HER2, and ErbB3/HER3, which in turn are responsible for signal transduction within the tumor cell [207, 209]. The PI3K-PKB pathway and extracellular signal-regulated kinase 1 and 2 (ERK1/2) mitogen-activated protein kinase (MAPK) are activated, and are responsible for the all properties of NT described in the following sections of this article.

**Figure 5:**
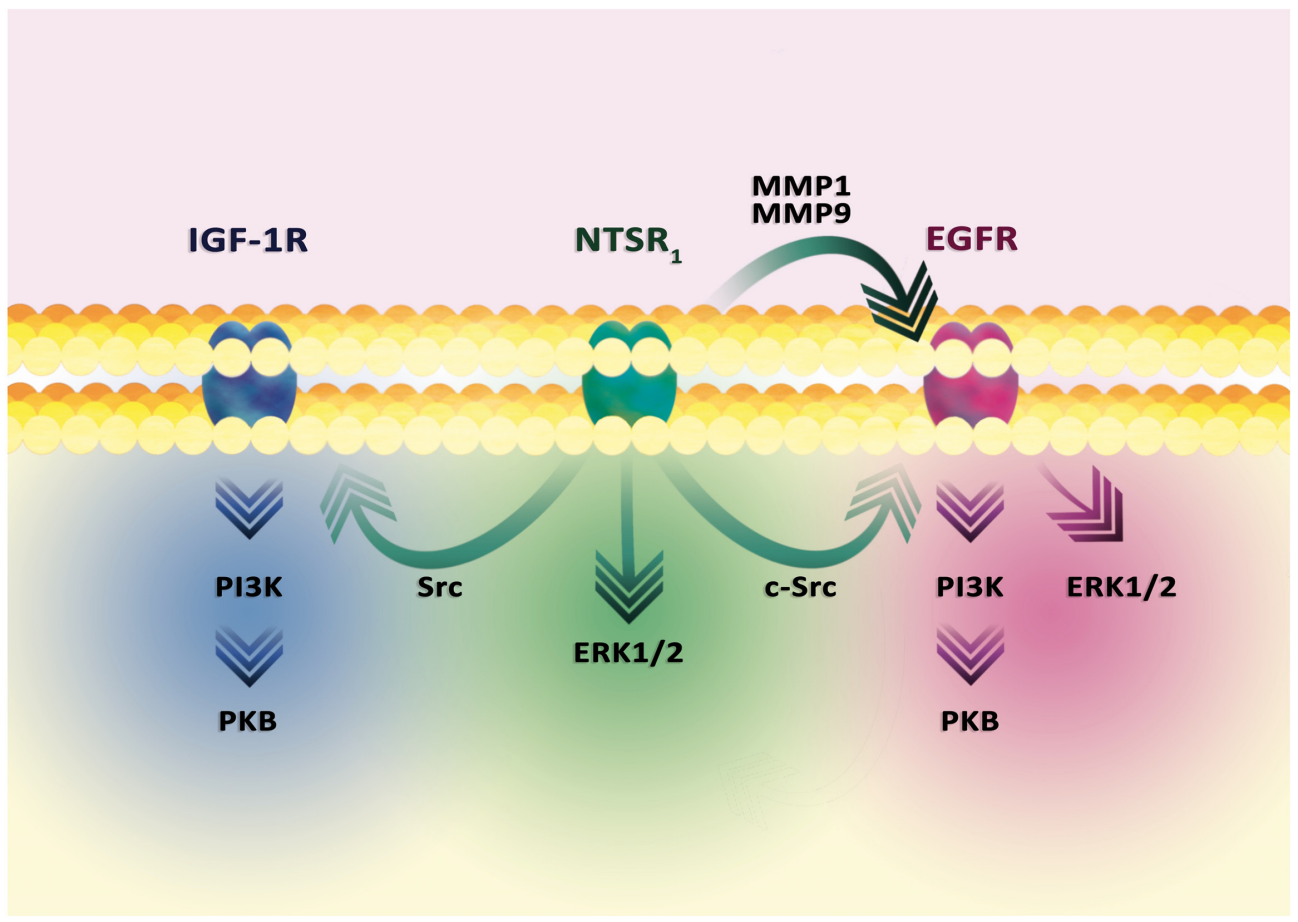
Signal transduction in the tumor cell from the NTSR_1_ receptor In general, the activation of NTSR_1_ leads to activation of ERK1/2 MAPK and PI3K-PKB cascades. The signal transmission involves EGFR, activated via c-Src. EGFR can also be activated by MMP1 and MMP9. These metalloproteins release the EGF-like ligands, thus activating these receptors. As a result, the ERK1/2 MAPK cascade and the PI3K-PKB pathway are activated. Nevertheless, the ERK1/2 MAPK cascade can be directly activated by the NTSR_1_-PLCβ-PKC pathway, without the involvement of other receptors. Similarly, the PI3K-PKB pathway can be activated by signal transduction to IGF-1R.

Activation of the EGFR family by NTSR_1_ is dependent on the PLC-β-protein kinase C (PKC) pathway, which increases expression and activates MMP1 and MMP9 (Figure 5) [207, 209–212]. In particular, the increase in MMP9 expression is responsible for PKC activation of the PI3K-PKB and ERK1/2 MAPK pathways [212]. MMP1 and MMP9 release epidermal growth factor (EGF)-like ligands, in particular heparin-binding EGF-like growth factor (HB-EGF), neuregulin 1 and neuregulin 2, which activate the EGFR family [209, 210]. As a result, these receptors activate ERK1/2 MAPK and the PI3K-PKB pathway [207].

EGFR activation may occur in a different manner. In prostate tumor PC3 cells, NT causes EGFR activation via c-Src [213]. This kinase causes phosphorylation of Tyr^845^ EGFR which results in STAT5b activation. Also, activated NTSR_1_ causes NF-κB activation which results in the increased expression of miR-21 and miR-155 [214]. miR-21 inhibits PTEN expression, a phosphatase degrading the PKB activator, which allows NT to increase the activity of this kinase. miR-155 reduces expression of the protein phosphatase 2 catalytic subunit α (PPP2CA), the suppressor of PKB activity.

There is also an EGFR-independent mechanism of signal transduction from NTSR_1_, i.e. via the activation of the PLCβ-PKC-ERK1/2 MAPK pathway. PI3K-PKB is also activated [207], which may involve another receptor with tyrosine kinase activity. An example of this is the insulin-like growth factor 1 receptor (IGF-1R) activated by Src in human colonic epithelial NCM460 cells [215].

GBM cells have been shown to express ErbB/HER2 while ErbB3/HER3 is more abundant on GSC [216–218]. Expression of these receptors, as well as the importance of EGFR amplification [81] in tumor processes in GBM, gives strong evidence that NT also acts through these receptors in this type of tumor.

### Neurotensin and glioblastoma stem cells

**Figure 6:**
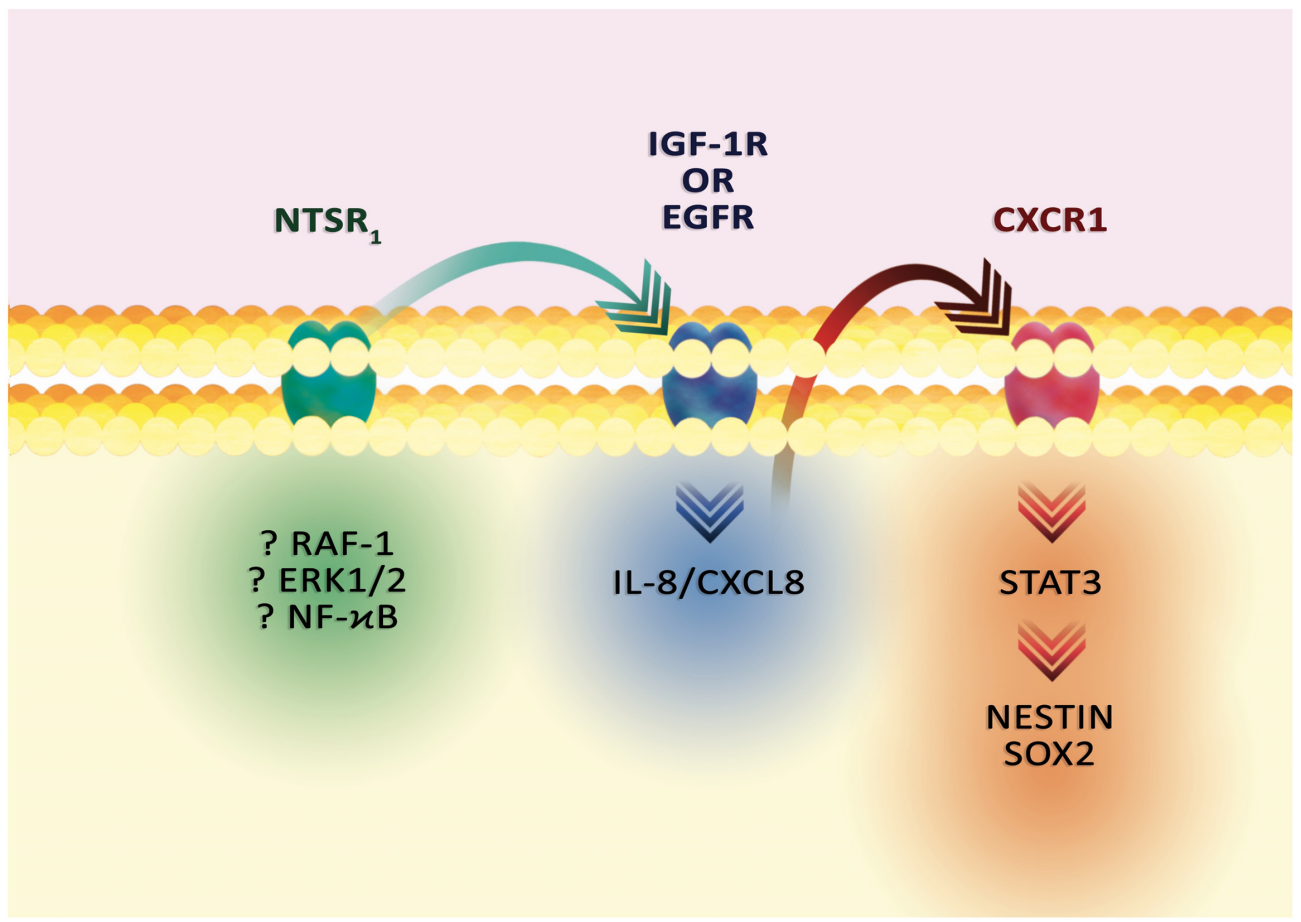
The effect of NT on GSC markers Activation of NTSR_1_ results in signal transmission to IGF-1R or EGFR and increased IL-8/CXCL8 expression. Then, the activation of CXCR1, an IL-8/CXCL8 receptor, activates STAT3 and increases the expression of stem cell markers: nestin and Sox2.

Expression of NT as well as receptors of this hormone in GBM occur mainly in GSC (Figure 6) [203, 219]. NTSR_1_ regulates the carcinogenic properties of GSC of various cells lines. The exact mechanism of NT effect on GSC is dependent on IL-8/CXCL8 [203]. NT after activation of NTSR_1_ and EGFR increases expression of IL-8/CXCL8 in GSC. Following the secretion of IL-8/CXCL8, this chemokine activates the CXCR1 receptor in an autocrine manner, which activates the STAT3 transcriptional factor. As a consequence, the expression of stem cell markers increases, especially nestin and Sox2, and sphere-forming ability is increased [203]. IL-8/CXCL8 also supports proliferation, migration and invasion [220]. Also, this chemokine is involved in angiogenesis and tumor immunosuppression [221, 222].

The exact mechanism of increased expression of IL-8/CXCL8 by NT in GBM has not been well understood. Experiments on other types of cancer show that ERK1/2 MAPK cascade, in particular ERK1/2 and RAF-1, are important in signaling, as demonstrated by stem cells of hepatocellular carcinoma and HCT116 human colorectal cancer [208, 223]. Also, the effect of NT on IL-8/CXCL8 expression may depend on NF-κB activation as demonstrated by transfected NCM460-line colonocytes and HCT116 human colorectal cancer [223, 224]. Nevertheless, the expression of IL-8/CXCL8 may also be activated by other receptors other than the EGFR family. In particular, the Src activation of the IGF-1R receptor can activate PKB, which increases expression of IL-8/CXCL8 in the colonic epithelial NCM460 cells [215].

### Effect on proliferation

NT stimulates the proliferation of GBM cells [202]. Activation of NTSR_1_ enhances the expression of CDK4 and CDK6 [204]. This effect is associated with a decrease in miR-129-3p expression and a reduction in miR-29b-1 expression via the NTSR_1_-c-myc pathway. These miRNAs reduce the expression of CDK6. This makes NT proliferate via the increased expression of CDK6. Also, NTSR_1_ via c-myc increases expression of CDK4 [204]. Thanks to these pathways, NT stimulates GBM cells to cross the G_1_/S checkpoint. In addition to the effects on proliferation, NT inhibits apoptosis by increasing Bcl-2 expression, as demonstrated on breast cancer MCF-7 cells [225].

### Effect on glutamate concentration

One of the features of gliomas, including GBM, is increased glutamate concentrations in the tumor environment [133]. This causes a neurotoxic effect. However, the impact of NT in this process is controversial. In the brain, NT increases glutamate concentration [199–201]. However, the induction of expression and activation of NTSR_1_ on GBM C6 cells results in an increase in the amount of excitatory amino acid carrier 1 (EAAC1) on the cell membrane, resulting in the uptake of aspartate and glutamate [226]. The involvement of NT in the neurotoxic effects of glutamate within GBM and gliomas requires further research.

### Effect on dissemination

NT stimulates GBM cells to invasion [202]. NT causes changes in the cytoskeleton organization. In particular, by activating Rac1 and cell division cycle 42 (Cdc42) increases the motility of U373 cells on laminin substrate [206]. Cells cultured on a plastics-only medium had more fibrillar actin and filopodial protrusions, and so showed lower motility [206].

Information on the effect of NT on GBM dissemination is incomplete, as no specific inhibitor studies have been conducted. Studies on other types of cancer show that NT acts via NTSR_1_ and NTSR_3_/sortilin which caused epithelial-mesenchymal transition [193, 212, 227, 228]. NT via NTSR_1_ in lung cancer cell lines NCI-H1299 activates FAK resulting in cell migration [229]. Also the migration of tumor cells is enhanced by sNTSR_3_ [192, 193]. Irrespective of NT or signal transduction from EGFR, NT increases FAK phosphorylation and activation [192]. Also, sNTSR_3_ decreases the expression of integrins, E-cadherin localization disorder, and changes in the desmosome structure, resulting in tumor cell release and migration [193]. Importantly, sNTSR_3_ does not affect tumor cell proliferation as it does not activate ERK1/2 MAPK [192].

In addition to FAK activation and changes in cytoskeleton organization and expression integration, NT also affects GBM dissemination by other means. In particular, NT induces an increase in IL-8/CXCL8 expression. By the action of this chemokine, the expression of MMP or activity of the uroplasminogen activation system is enhanced, as was the case in pancreatic adenocarcinoma BxPC-3 and PANC-1 lines [230].

### Neurotensin and angiogenesis

To date, the effect of NT on angiogenesis has not been unambiguously determined. However, it may be inferred that – similar to immune evasion – it is cell-specific. In experiments on human umbilical vein endothelial cells (HUVEC) NT has not been reported to cause angiogenesis [231]. However, in experimental colitis of the large intestine, NTSR_1_ is a factor that does enhance angiogenesis [232]. In particular, NTSR_1_ activity stabilizes and increases HIF-1α expression. This results in increased expression of genes dependent on this protein, especially VEGF-A which is involved in angiogenesis [232].

NT can also indirectly influence tumor angiogenesis through IL-8/CXCL8. NT induces an increase in IL-8/CXCL8 expression in GSC [203]. This chemokine causes the recruitment of tumor-associated neutrophils which secrete various hormones involved in angiogenesis [222, 233].

### Neurotensin and tumor immune evasion

#### Effect on macrophages

The effect of immunological processes is cell-specific. NT does not affect macrophages in tumor immune evasion but instead enhances the already induced immune response by increasing macrophage activation, although this effect is about 10 times smaller than at 100 U/ml IFN-γ [234–236]. In experiments on RAW 264.7, NT does not affect migration, expression of TNF-α, IL-10, nor IL-12 in macrophages [236]. In contrast, NT increases IL-1β and IL-6 expression [236]. NT also strengthens the immune response as demonstrated on lipopolysaccharide (LPS)-activated RAW 264.7 macrophages [236] and rat alveolar macrophages [237]. NT increases the production of TNF-α, IL-1β and IL-12 [236]. However, NT does not modify the expression of IL-1 caused by IFN-γ nor that caused by leukotriene B_4_ [237]. NT also increases the migration and expression of COX-2 and inducible nitric oxide synthase (iNOS) in LPS and IFN-γ activated macrophages [235]. The NT effect on macrophages is associated with NTSR_1_ activation, which affects LPS-activated NF-κB factor and the activated JAK2-STAT1 pathway [235]. However, due to immunosuppressive processes in tumors [238], NT does not directly play any direct role in the activation or migration of macrophages in GBM tumors.

#### Effect on microglial cells

In a similar way NT affects microglia. This is a heterogeneous population of cells; with only 8% of adult mouse brain cells and 13% of neonatal C57Bl/6 mouse brain reacting to NT [239]. Activation of microglial cells of these populations, especially in the neonatal brain of mice, may be impaired by the action of a previously anti-inflammatory cytokine such as IL-4 [239]. Nevertheless, the number of NT-responsive microglial cells may increase. If adult mouse brain microglial cells are activated with a proinflammatory cytokine such as IFN-γ, the number of NT-responsive cells increases 3 times [239]. This effect does not occur under the influence of LPS or IFN-γ on microglia isolated from a neonatal mouse brain.

Microglial cells express NTSR_3_/sortilin but not NTSR_1_ [240–242]. Activation of this receptor causes migration of microglial cells. This effect is triggered by the activation of PI3K and MAPK cascades [240]. This results in changes in F-actin polymerization and filopodia formation. Via NTSR_3_/sortilin activation, NT induces an increase in the expression of IL-1, TNF-α, CCL2/MCP-1, CCL5/RANTES, IL-8/CXCL8, CXCL2/MIP-2 but not altering the expression of IL-6, CCL3/MIP-1α nor CCL4/MIP-1β [241, 242]. The effect on the expression of these hormones is dependent on the activation of ERK1/2 MAPK and PI3K in microglial cells [241]. Chemokines produced by microglial cells participate in the migration and recruitment of other microglial cells (CCL2/MCP-1 and CCL5/RANTES) [134] as well as macrophages (CCL2/MCP-1 and CCL5/RANTES) [134, 136, 146], neutrophils (IL-8/CXCL8, CCL2/MCP-1 and CXCL2/MIP-2) [139, 140] and T_reg_ (CCL2/MCP-1) [129]. In contrast, pro-inflammatory cytokines enhance the immune response. Nevertheless, the immunosuppressive tumor microenvironment decreases the number of NT-responsive microglial cells [238].

#### Effect on neutrophils

NT also has an effect on neutrophils. However, this action is poorly understood and requires further investigation in GBM tumors. It has been shown that *in vitro* NT at a concentration as low as 0.1nM acts as a chemoattractant for neutrophils, increasing their targeted migration [243]. This effect is direct via NT receptors on these cells and also indirect via IL-8/CXCL8 [203, 222]. NT expression, occurring predominantly in GSC [203, 219], may explain the accumulation of neutrophils in the GBM tumor near the GSC [185]. Further studies on the effect of NT on the migration of neutrophils to the tumor niche and the location of these cells in the tumor are required.

After the migration of neutrophils near GBM, NT increases adherence and diapedesis of neutrophils, thereby increasing the infiltration of these cells within the tumor. This has been demonstrated by *in vitro* experiments in which NT caused adherence to bronchial epithelial cells [244]. It seems that NT induces neutrophil activation, in particular phagocytosis of these cells [243].

#### Effect on dendritic cells

NT has immunosuppressive properties on fetal-skin dendritic cells [245]. NT reduces the production of TNF-α, IL-10 and VEGF in these cells, which is anti-angiogenic [245]. On the other hand, NT enhances the synthesis of EGF in these cells, which may have a significant effect on GBM with amplified EGFR near dendritic cells. NT also interferes with LPS activity in dendritic cells. Incubation of NT together with LPS or pre-incubation of NT completely abolishes dendritic cell response to LPS, in particular the expression of TNF-α, IL-6 and IL-10 [245]. This shows that NT can interfere with the antitumor immune response.

## GROWTH DIFFERENTIATION FACTOR-15 / MACROPHAGE INHIBITORY CYTOKINE-1

### Growth differentiation factor-15 as an antineoplastic agent

GDF-15/macrophage inhibitory cytokine-1 (MIC-1) is a member of a TGF-β superfamily hormone [246]. To date, no receptor for this extracellular protein has been identified [247, 248]. GDF-15 seems to act via the TGF-β receptor type II (TGFβRII) [249, 250]. Heterodimers of TGFβRI/activin receptor-like kinase (ALK-5) and TGFβRII are also important in the signal transduction. According to a recent study, the GDNF family receptor α-like (GFRAL) is a specific GDF-15 receptor [251–253]. Thanks to this, overexpression of GDF-15 results in decreased appetite and weight loss [254, 255]. GDF-15 is also associated with the cachexia associated with cancer. This property of GDF-15 may explain the decreased appetite and drastic weight loss in some glioma and GBM patients [256].

GDF-15 plays a very important and diverse function in cancer processes. At the beginning, it has antitumor properties, as it inhibits tumor cell division (Table 1). It induces phosphorylation of Smad3 (a protein that participates in tumor suppression) and apoptosis via the intrinsic mitochondrial pathway, as demonstrated on U-87 MG, U-118 MG, U-251 MG, U-373 MG and T98G cell lines [248, 257]. GDF-15 also disrupts connective tissue growth factor (CTGF)-induced angiogenesis in HUVEC [258]. In particular, GDF-15 decreases FAK activation and decreases clustering of the α_V_β_3_ integrin. Expression of GDF-15 is enhanced by the action of the p53 protein in an antitumor mechanism [259]. However, the resulting mutations in the *P53* gene and hypermethylation of the *GDF15* gene promoter result in reduced expression of this protein in cancer cells [257]. In GBM cells the epigenetic silencing of Egr-1 and Sp-1 transcription factors results in a decrease in expression of GDF-15, as demonstrated with the use of histone deacetylase inhibitor Trichostatin A [260].

**Table 1:** Anti- and pro-cancer properties of GDF-15

	Anti-cancer properties	Pro-cancer properties
Cell viability	Inducted intrinsic mitochondrial apoptosis pathway	Increased viability via increased activation of the PI3K-PKB pathway
		Neutralization of the cytotoxic action of TGF-β1
Angiogenesis	Reduction in CTGF-dependent angiogenesis	In hypoxia, an increased expression of HIF-1α and VEGF
Migration and invasion	Decreased FAK activity	Increased expression of MMP2 and MMP9
	Decreased intensity of integrin α_V_β_3_clustering	Inducted epithelial-mesenchymal transition
Tumor immune escape		Reduced IL-2 synthesis
		Increased IL-10 synthesis
		Reduced IL-12 synthesis
		Increased TGF-β1 synthesis
		NK dysfunction
		Reduced infiltration of macrophages and T cells.

### Growth differentiation factor-15 as a progression of cancer

As cancer progresses, tumor cell resistance to GDF-15 and its elevated synthesis increases [261, 262]. This is an indication of the progression of cancer; hence a high correlation of this hormone level and a reduction in survival of patients after GBM removal [262, 263]. The expression of GDF-15 in secondary glioblastomas is much higher than in primary glioblastomas [261]. Nevertheless, expression of GDF-15 is not the same for all GBM cells in the tumor. The highest expression occurs in the mesenchymal subtype, with the lowest in the proneural subtype [262]. Another significant source of GDF-15 are TAM, as shown in esophageal squamous cell carcinomas [264].

Related to the progression of tumor formation, resistance to GDF-15 is associated with changes in the pathways activated by this hormone. In particular, GDF-15 no longer causes Smad3 phosphorylation [263]. As a result, the hormone no longer causes apoptosis of the GBM lines, particularly lines A172 and LN-229 [261–263, 265]. These lines, unlike the U-87 MG and T98G lines (not-resistant to GDF-15), have a high expression of this protein [261, 262, 265].

It has also been shown that GDF-15 stimulates the intensity of proliferation not only of LN-229 and A172, but also of LN-319, U-87 MG, D-247 MG, LN-308, LN-428, LN-18 and U-373 MG [261]. On the other hand, studies by Kadowaki et al. and Zhang et al. show an opposite effect. GDF-15 reduces the proliferation of U-87 MG and U-373 MG cells lines [248, 257]. In addition to Smad3 pathways, GDF-15 also increases the activation of the PI3K-PKB pathway which increases the viability of the cells [248]. Another pathway of GDF-15 proliferative activity is TGF-β1 dysfunction [266]. Accumulated in the cancer cell nucleus, GDF-15 causes a disorder in the expression of genes associated with Smad factors [266]. Smad factors are associated with the transduction of a signal from the TGF-β1 receptor. In this way, GDF-15 abolishes the inhibitory effect on cell division TGF-β1.

#### Effect on angiogenesis

In addition to proliferation, GDF-15 also induces angiogenesis in advanced tumor processes. GDF-15 stimulates the proliferation of HUVEC via increased expression of cyclins D1 and E [267, 268]. This effect depends on the activation of PI3K-PKB and ERK1/2 and JNK MAPK pathways. In anoxia, expression of GDF-15 increases in GBM cells independently of p53 and HIF-1, as demonstrated in the LN-Z308 cell line [269]. Then, this hormone causes angiogenesis in hypoxic conditions which corresponds to increasing peritumoral angiogenesis in region of raised regional tissue tension followed by regional cerebral blood flow failure causing hypoxia [270]. Through the stabilization of the p53-MDM2 complex, it disrupts the p53 function in vascular cells [271]. This is followed by an increase in HIF-1α expression and an increase in VEGF expression, as demonstrated in HUVEC [271]. In hypoxia, this causes angiogenesis in the tumor.

#### Effect on migration and invasion

GDF-15 also promotes migration and invasion of GBM cells [262]. Anti-GDF-15 antibodies induce a decrease in the invasive capabilities of lines such as U-373 MG and LN-308 while increasing the invasion capability of the LN-428 line [261]. This indicates that depending on tumor cell changes, GDF-15 inhibits or enhances the migration and invasion of GBM cells. Nevertheless, GBM is a tumor with high intratumoral heterogeneity. As a result, this cytokine can cause the migration of certain tumor lines sensitive to GDF-15 in the GBM tumor. Linking such changes in the cancer cell to the effect of GDM-15 requires further investigation. It is known that GDF-15 affects the activity of the uroplasminogen activation system in LNT-229 and LN-308 glioma cells. GDF-15 induces an increase in miRNA expression, silencing plasminogen activator inhibitor-1 (PAI-1), and a less pronounced silencing ofthe expression of urokinase-type plasminogen activator (uPAR) receptor [262]. More precise studies have shown that GDF-15 affects GBM cell migration independently of uroplasminogen activation system expression [262].

GDF-15 can have an affect on invasion by using other pathways as demonstrated in experiments using other cancers. GDF-15 causes epithelial-mesenchymal transition (EMT) of colorectal cancer in HT29 and SW480 cell lines [272] and carcinoma cell line HepG2 [273]. GDF-15 results in decreased expression of E-cadherin and increased expression of N-cadherin and vimentin.

The adhesion of GBM cells is performed by integrins [274]. GDF-15 may interfere with integrin activation [250, 275]. However, the impact on GBM migration via this pathway still needs to be investigated further. This cytokine may also increase the expression of MMP2 and MMP9 by activation of the PI3K-mTOR pathway, as demonstrated in ovarian cancer cells [276]. However, the impact on GBM via this pathway is yet to be confirmed.

#### Effect on tumor immune escape

GDF-15 also causes tumor immune escape. In experiments on splenocytes, it reduced IL-2 synthesis and increased the synthesis of immunosuppressive IL-10 [265]. GDF-15 has been shown to impair NK function and reduce malignant infiltration of macrophages and T cells in tumors [265]. In addition, GDF-15 causes dendritic cell function abnormalities. It reduces the synthesis of IL-12 and increases the synthesis of TGF-β1, a cytokine that also strongly disrupts the immune function [277]. GDF-15 also decreases the expression on dendritic cell membrane proteins, particularly CD25, CD83, CD86 and HLA-DR [277, 278]. These changes cause disorders in the stimulation of the antitumor immune response, in particular a reduction in the stimulation of cytotoxic T lymphocytes and other immune cells [277]. This causes immunosuppression in the tumor microenvironment.

In addition to silencing the antitumor immune response, GDF-15 affects cell migration to the tumor niche. In particular, it enhances expression of CCL2/MCP-1 chemokine via TGFβRII-SMAD-3, as demonstrated on RAW 264.7 macrophages [249]. GDF-15 action via TGFβRII differs from the activation of this receptor by TGF-β (which does not increase the expression of CCL2/MCP-1). In addition to its effects on chemokine expression, GDF-15 exhibits increased expression of CCL2/MCP-1 receptor (CCR2) in macrophages [249]. This cytokine also changes CCR2 phosphorylation, which increases the intensity of activation of this receptor [249].

GDF-15 may interfere with the recruitment of monocytes and neutrophils into the tumor niche. In particular, GDF-15 disrupts integrin activation on THP-1 monocytes and murine neutrophils [250, 275]. This results in abnormal adherence and diapedesis of these cells and hence a decrease in infiltration of monocytes and neutrophils to other tissues. This effect is dependent on TGFβRI/ALK-5 and TGFβRII receptors [250].

## SPHINGOSINE-1-PHOSPHATE

### Sphingosine-1-phosphate synthesis, degradation, and receptors

Sphingosine-1-phosphate (S1P) is a hormone; a sphingolipid synthesized from sphingosine by two sphingosine kinase isoforms: sphingosine kinase 1 (SphK1) and sphingosine kinase 2 (SphK2) [279, 280]. These enzymes catalyze the same reaction but have different cellular locations and functions [280]. Activated SphK1 attaches to the cell membrane and catalyzes the formation of S1P; it is responsible for the concentration of S1P outside the cell. The activity and product of the reaction catalyzed by this enzyme have antiapoptotic and promitogenic properties. In contrast, SphK2 is primarily a nuclear enzyme. Its inactive form is attached to biological membranes (in particular to the cell membrane) and to the endoplasmic reticulum via the BH3 domain. SphK2 activity has proapoptotic properties and inhibits cell division.

S1P is inactivated in two ways. First, it can be dephosphorylated by S1P-catalyzed phosphohydrolase (SPP)1 or SPP2. Another way to inactivate this hormone is through breakdown by S1P lyase (SPL).

The synthesized S1P can act as a second messenger, as well in an autocrine or paracrine manner via S1P receptors on the surface of cells. As the second messenger, S1P activates peroxisome proliferator-activated receptor γ (PPARγ) and thus performs important functions in HUVEC physiology [281]. However, S1P is also secreted outside the cell. Then, in an autocrine or paracrine manner it activates five of its receptors (S1PR_1-5_), coupled with different small G proteins and thus differing in signal transduction and function [282].

### Sphingosine-1-phosphate-related enzymes in the glioblastoma multiforme tumor

S1P plays a very important role in apoptosis [283–285], homeostasis of the immune system [284, 286] and blood vessel physiology [287]. An increasing number of papers show that S1P plays a very important role in the pathogenesis of cancers, including the development of brain tumors. GBM is associated with the overexpression of S1PR_1_, S1PR_2_, S1PR_3_ and S1PR_5_ and higher S1P concentrations than in the rest of the brain [288, 289]. At the same time, S1PR_4_ is not expressed in this tumor or in normal brain tissue [288, 289].

In contrast, SphK1 expression is higher in recurrent and secondary GBM, whereas SphK2 is higher in primary GBM [288]. Also, the expression of S1PRs differs in GBM. The expression of S1PR_1_ and S1PR_5_ is elevated in all types of GBM, mostly in secondary GBM [288]. In contrast, increased expression of S1PR_2_ and S1PR_3_ occurs only in secondary GBM [288].

A reduction in S1PR_1_ expression is associated with a shorter postoperative survival time of patients [289–291]. Also the overexpression S1PR_2_ [289], S1PR_5_ [288] SphK1 [292–294] and SPP1 [289] is associated with short postoperative survival time. Nevertheless, different studies indicate different proteins related to the survival of patients. Research by Bien-Möller et al. shows that the expression of S1PR_3_, S1PR_5_ and the enzymes SphK1, SphK2, SPP2 and SPL1 is not related to survival [289]. In contrast, Quint et al. show that S1PR_1_, S1PR_2_, S1PR_3_, SphK1 and SphK2 have no such effect [288].

### Effect on glioblastoma cell viability

*In vitro* experiments show that S1P and enzyme expression involved in the biochemistry of this hormone influence the viability and behavior of GBM cell lines. The induction of expression and activity of SphK1 are influenced by various factors, in particular activation of the receptors of PDGFR [295] EGFR [296], and the expression of variant III of EGFR mutation (EGFRvIII) [294]. These receptors are closely involved in the development of GBM [80, 81, 297].

In addition to growth factors, inflammatory reactions also increase the expression of SphK1. IL-1 enhances the expression of SphK1 in GBM cells via c-Jun terminal kinase (JNK) MAPK and AP-1, independently of NF-κB [298]. Hypoxic stress also increases the expression of SphK1 [299, 300]. It increases the expression and activity of SphK1 and thus the extracellular concentration of S1P. SphK1 increases the rate of cell proliferation, increases migration and invasion, and inhibits multiple glioma cell lines, in particular LN-229, LN-382, U-87 MG, U-373 MG, U-1242 MG, and primary human non-established GBM GBM6 cells [292, 301, 302]. In particular, the activation of PKB by S1PRs results in inactivation of FOXO3a and consequently a decrease in the expression of proapoptotic Bcl-2-like protein 11 (Bim) [302]. The expression of S1PRs, mostly S1PR_1_, increases the rate of proliferation of U-118 MG and U-373 MG cells [303]. This effect is related to the activation of ERK1/2 MAPK. S1PR_5_ has been shown to inhibit proliferation by inhibiting ERK1/2 MAPK activation [303]. Nevertheless, Yoshida et al. showed the opposite results, with S1PR_1_ decreasing tumor cell proliferation rates in U-87 MG and U-251 MG cancer cell lines [291].

S1P has different effects on different cell lines. LN18 cell proliferation is not affected by signal transduction from any of the S1PRs [289]. This is due to mutations in the *P53* gene, which results in the independence of proliferation and apoptosis of cells with mutations in that gene from the level of S1P [304].

### Effect on glioblastoma stem cells

**Figure 7:**
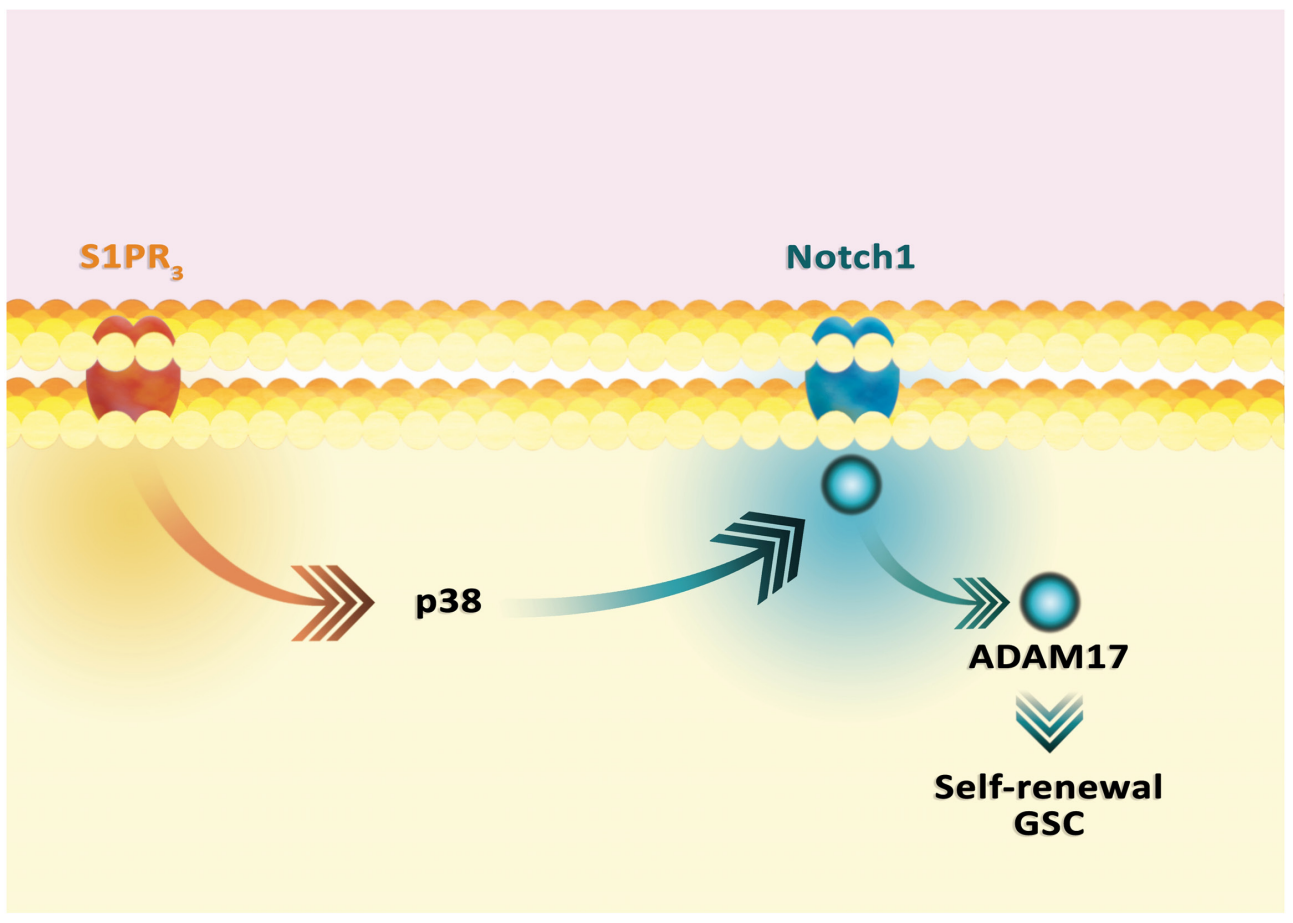
Activation of Notch1 pathway by S1P The activation of S1PR_3_ activates the Notch1 pathway. This activation is independent of the Notch1 ligand and is dependent on the ADAM17 protein, which results in GSC self-renewal.

The action of S1P differs according to the cell type. The greatest synthesis and secretion of S1P occurs in GSC [305]. The expression of S1PR_1_, S1PR_2_ and S1PR_4_ also occurs predominantly in these cells, which results in the fact that it is GSC that mainly react to S1P [306]. For example, S1P in an autocrine manner increases the life span of the GSC. It stimulates the expression of GSC markers [307]. S1P also stimulates GSC proliferation and has antiapoptotic and antinecrotic effects [307]. As a result of this action, S1P causes resistance of GSC to TMZ, which is independent of methylguanine-methyltransferase (MGMT) expression [305, 308]. These S1P properties are due to the Notch pathway in GSC [309], crucial because of its role in promoting proliferation and self-renewal of these cells in the GBM tumor (Figure 7) [310]. Signal transduction from S1PR_3_ induces the p38 MAPK-dependent ADAM17 activation in the signal transmission from Notch1. This ADAM17 activation mechanism is independent of Notch1 receptor activation.

### Hypoxia and angiogenesis

S1P also participates in angiogenesis. In the model of hypoxia using CoCl_2_ in GBM cells, the expression of SphK2 decreased and expression of SphK1 increased [299, 300]. This results in an increase in the synthesis and concentration of S1P in the tumor microenvironment [299]. The increase in SphK1 expression was due to an increase in HIF-2α activity that binds to the promoter of the SphK1 gene [299]. HIF-1α likewise has this effect. Increasing S1P concentrations stimulates GBM cells to proliferate and inhibit apoptosis by ERK1/2 MAPK and PKB activation [300, 302, 303].

S1P not only affects tumor cells in an autocrine manner but also blood vessel cells. In particular, it initiates endothelial cell sprouting and migration, and formation of ‘tubes’ as shown on HUVEC, human dermal microvascular endothelial cells, and mouse embryonic fibroblasts [299, 311, 312]. This process is mainly triggered by S1PR_1_ activation under hypoxic conditions [299].

However, in HUVEC, S1P may participate in angiogenesis independently of its specific receptors. S1P in these cells directly activates PPARγ, resulting in an increase in PAI-1 expression [281]. Importantly, S1P does not affect the expression of proteins involved in lipid metabolism. A consequence of PAI-1 expression is the stimulation of angiogenesis [313].

The endothelial structures formed by cells are not stable, and so S1P is not a sufficient factor for the entire course of angiogenesis. It is other proangiogenic factors that support the formation of new vessels in the tumor [311]. That is why in hypoxia VEGF expression occurs later than SphK1 expression [299]. Nevertheless, it seems that VEGF and S1P work together in angiogenesis, mutually enhancing each other's action [299, 314].

### Effect on migration and invasion

GBM is a tumor that always recurs after surgery. The migration and invasion of GBM cells depends on several factors, including S1P and the proteins involved in the biochemistry of this hormone. The expression of S1PR_1_ and S1PR_3_ is responsible for the migration and invasion of U-118 MG and U-373 MG cells [303]. This process depends on the plasminogen activation system. In particular, in A172, U-118 MG, and U-373 MG cells, the overexpression of S1PR_1_, and to a lesser extent that of S1PR_2_ and S1PR_3_, results in an increase in urokinase-type plasminogen activator (uPA) and uPAR activity, independently of S1P [293, 315]. In contrast, in U-373 MG cells the overexpression of S1PR_1_ and S1PR_2_, as well as activation of S1PR_2_ by S1P, result in increased expression and activity of uPAR and PAI-1 [293, 315, 316]. This is due to the activation of MEK1/2 and Rho by S1PRs [316]. This signal transduction also involves protein kinase D2 (PKD2) [317]. The activation of S1PRs activates PKD2. As a consequence, GBM cells express the proteins involved in migration and invasion, in particular proteins associated with plasminogen, integrin α-2, integrin α-4, and MMP1 [317].

S1P-dependent GBM cell migration and invasion is influenced by other signaling pathways; in particular EGFR-Src-PKCδ activates SphK1 [315]. As a consequence, PAI-1 is expressed in U-373 MG and A172 cells [315]. S1P-dependent expression of PAI-1 and uPAR may also be enhanced by IL-1 [316].

In some models, S1PR_1_ and S1PR_2_ activation inhibits migration but increases invasiveness by increasing the adhesion of U-87 MG, U-118 MG, U-251 MG and U-373 MG cells [293, 303, 318]. This process depends on the induction of cysteine-rich angiogenic inducer 61/CCN family member 1 (Cyr61/CCN1) expression [293, 303].

Another very important route in the migration and transfiguration of GBM cells by S1P is Ca^2+^ mobilization [295, 296, 306]. Activation of S1PRs results in signal transductions involving MAPK, RhoA/ROK, and phospholipase C [296]. This signaling also involves membrane-type-1 MMP and the glucose-6-phosphate transporter; the silencing of their expression impairs the effect of S1P on Ca^2+^ mobilization [296].

The migration of GBM cells via Ca^2+^ mobilization may partly depend on cytoplasmic S1P, i.e. independently of S1PRs [295]. In particular, S1P activates transient receptor potential C1 (TRPC1), which causes Ca^2+^ mobilization in the cytoplasm [295]. In U-252 MG cells, this mechanism is induced by PDGFR activation, in particular the synthesis of S1P.

### Tumor immune evasion: effect on macrophages

In GBM, TAM play an important role in immune modulation and tumor development [75]. The amount of TAM increases with the grade of the glioma [319]. Thus, they constitute a significant percentage of cells in the GBM tumor [176, 320, 321].

There are currently no studies showing the effects of S1P from glioma or GBM on TAM. Nevertheless, on the basis of work on melanoma [322], breast cancer [323] and S1P biochemistry research in GBM, it can be deduced that this hormone significantly influences macrophage behavior in brain tumors particularly angiogenesis [292–294].

No sudden angiogenesis occurs during a growth in tumor volume. Angiogenesis is only induced by hypoxia and signaling pathways activated by tumor microenvironment [324]. Cell apoptosis occurs very often in tumor microenvironment. Apoptotic bodies contain S1P produced by SphK2, which affects macrophages [325–329]. However, SphK1 may also be activated during apoptosis, especially during the action of antitumor drugs [283]. S1P in apoptotic bodies causes large changes in macrophages; it activates S1PR_1_, which results in an increase in HIF-1α expression even in normoxia [328]. As S1P alone does not increase the expression of HIF-1α, in order to induce a given effect the apoptotic bodies also activate other non-S1P signaling pathways. With regard to the effect of S1P on HIF-1α expression, the second factor is TGF-β. An increase in HIF-1α expression activates mechanisms leading to angiogenesis in the tumor.

SphK activity is not important in the differentiation of progenitor cells into monocytes [330]. In contrast, S1P is important in the egress of monocytes from the spleen and bone marrow, as demonstrated by the use of FTY720. This process is dependent on S1PRs, with the exception of S1PR_3_ [331]. In monocytes already circulating in the blood, particularly in immunosuppressive mouse monocytes (CD45^+^CD11b^+^Gr1^−^), activation of receptors S1PR_2_ and S1PR_3_ by S1P activates PI3K and induces migration of these cells [332–334]. Activation of S1PR_1_ and S1PR_5_ does not result in the migration of monocytes [334, 335].

Targeted monocyte migration induced by S1P is partly dependent on thrombin. S1P induces increased expression of protease-activated receptor-4 (PAR-4), a thrombin receptor [336], which directs the migration of monocytes to the site of elevated thrombin activity. This receptor also increases expression of COX-2. Monocyte migration mediated by S1P and thrombin can exist within the GBM tumor because this cancer has elevated thrombin activity and increased activity of SphK1 and S1P levels [337].

Activating S1PR_1_ and S1PR_3_ on macrophages, S1P acts as a chemoattractant for these cells [283, 332, 338–340]. Activation of S1PRs in macrophages results in ADP secretion and the synthesis of extracellular ATP via adenylate kinase activity. In consequence, the P2X_7_ receptor is activated on macrophages [341]. This results in changes in actin polymerization, which facilitates migration. In contrast, S1PR_2_ activation reduces macrophage migration [338, 342], which is associated with increased cAMP levels and decreased PKB phosphorylation.

In addition to the effect on chemotaxis, S1P results in increased expression of intercellular adhesion molecule I (ICAM-1), which increases monocyte adhesion to these cells. This effect has been demonstrated on HUVEC, where ICAM-1 expression was dependent on S1PR_1_ [343]. S1P has also been shown to increase expression of E-selectin in HUVEC [344]. This effect depended on S1PR_1_ which activated PI3K-PKB and ERK1/2 MAPK pathways. It induced an increase in SphK activity with intracellular S1P playing the role of a second messenger [344]. Intracellular S1P activates NF-κB, which increases the expression of genes dependent on this transcription factor, including an increased expression of E-selectin [345–347].

S1P has also been shown to increase ICAM-1 expression on human pulmonary alveolar epithelial cells in a process mediated by S1PR_1_ and S1PR_3_ [348]. In these cells, ICAM-1 expression depended on the activation of ERK1/2 MAPK, p38 MAPK and JNK MAPK, and on c-Src kinase, EGFR, PDGFR and PKB [348]. However, S1P seems to disrupt the adhesion of monocytes to the walls of blood vessels [349]. It causes rearrangement on HUVEC integrins α_5_β_1_ and α_V_β_3_ cells, which impairs monocyte adhesion to these cells [350]. In addition to the effect on adhesion proteins, S1P increases the expression of chemokines that attract monocytes and macrophages. Via S1PR_1_ and S1PR_3_, S1P causes an increase in the expression of CCL2/MCP-1 in HUVEC [343]. An increase in the expression of other chemokines may also be involved in the S1P-induced migration of monocytes. CYM-5442, an S1PR_1_ agonist, reduces the expression of CCL2/MCP-1 and CCL7/MCP-3 in HUVEC [351].

Macrophages phagocytose the apoptotic bodies, which enables the removal of cells that are subject to apoptosis. S1P from apoptotic bodies activates S1PR_1_ on macrophages and inhibits their apoptosis [326]. This effect is dependent on the level of intracellular Ca^2+^ and the activation of ERK1/2 and PI3K. It increases the expression of Bcl-2 and Bcl-x_L_ and causes the phosphorylation of Bcl-2-associated death promoter (BAD) in these cells. High density lipoproteins (HDL), which contain S1P, also inhibit macrophage apoptosis [352]. In particular, S1PR_1_, S1PR_2_ and S1PR_3_ are activated, which results in the activation of the STAT3-JAK2 pathway and therefore an increase in survivin expression.

Nevertheless, the phagocytosis itself also partly depends on S1P. S1PR_2_ reduces the intensity of *Escherichia coli* phagocytosis by increasing the RhoA-GTP level. This causes a contraction of the macrophages [353]. S1PR_2_ reduces the amount of Rac1-GTP, which inhibits actin polymerization and therefore disrupts phagocytosis in macrophages [353]. However, S1PR_2_ stimulates antibody-dependent phagocytosis [354].

S1P reduces the pro-inflammatory immune response in macrophages. Via S1PR_1_, but not S1PR_2_, S1P reduces the expression of inflammatory cytokines from LPS-activated macrophages. In particular, it reduces the expression of IL-12, TNF-α and CCL2/MCP-1 and increases the expression of arginase I. Which enzyme reduces nitric oxide (NO) production in macrophages. S1P also reduces the activation of NF-κB in LPS-treated macrophages, which reduces the expression of iNOS [355].

Activation of macrophages by proinflammatory agents, such as LPS or TNF-α, results in increased production and secretion of proinflammatory cytokines. Nevertheless, SphKs do not participate in the signal transduction induced by LPS or TNF-α as demonstrated *in vivo* and *in vitro* using gene knockouts of these enzymes in murine monocytes and macrophages [330]. Similar results have been obtained on RAW 264.7 macrophages [356]. SphK1 does not affect the LPS-induced prostaglandin E_2_ (PGE_2_) production. On the other hand, increased SphK1 activity is required in RAW 264.7 to increase PGE_2_ production in response to TNF-α [356], which is related to S1P involvement as a second messenger in TNF-α–induced activation of NF-κB [345–347]. Then, after chemotaxis, this hormone silences excessive immune response in macrophages [285]. However, this property of S1P also plays an important role in cancer immune evasion [322, 323, 357]. S1P polarizes to M2 macrophages [322, 323, 326, 329], with a significant role of increased HO-1 expression in this process. HO-1 expression is due to the activation of two pathways by S1PR_1_: a p38 MAPK-dependent pathway and another which activates STAT1 and increases VEGF expression [329]. Then, an increase in HO-1 activity causes an increase in expression of antiapoptotic proteins in macrophages, in particular Bcl-x_L_ and Bcl-2 [329]. HO-1 also increases the expression of adenosine A_2A_ receptor in macrophages, one of the immunosuppressive mechanisms in immune cells [329].

S1P significantly changes the secretory profile of macrophages. In apoptotic bodies S1P increases the expression of COX-2 protein, in part by stabilizing COX-2 mRNA [356, 327, 358]. In addition to the effect on this enzyme, S1P also increases the activity of microsomal prostaglandin E synthase-1 (mPGES1) and decreases the activity of prostaglandin D synthase (PGDS) and 15-hydroxyprostaglandin dehydrogenase (15-PGDH). In the tumor microenvironment, this increases the production of PGE_2_, a compound which participates in many mechanisms of GBM development [359, 360]. S1P increases the expression of lipocalin 2 (LCN2) in macrophages, resulting in lymphangiogenesis in the breast tumor model [361].

S1P also influences NO production in macrophages. The activation of S1PR_2_ on macrophages by the apoptotic bodies [362] induces the activation of extracellular signal-regulated kinase 5 (ERK5) and subsequent activation of the cAMP responsive element binding protein (CREB). This, in turn, increases the expression of arginase II, an enzyme that metabolizes L-arginine, an amino acid that serves as a substrate for nitric oxide synthase. This results in a reduction in iNOS activity and NO production. ERK5 also increases expression of CD206 and VEGF. Independently of ERK5, activation of S1PR_2_ results in an increase in IL-10 expression [362].

As a result of exposure to S1P, indoleamine 2,3-dioxygenase (IDO), IL-8/CXCL8, IL-10 and CD206 expression increases, NO levels decrease, TNF-α and IL-12 expression decreases, i.e. polarization to M2 macrophages occurs [284, 322, 323, 326, 329].

In resident peritoneal macrophages, S1P reduces the production of poinflammatory cytokines via S1PR_2_, as has been demonstrated on a model with an abnormal activity of this receptor. Knockout of Gα_12/13_, a significant protein in the transduction of signals from S1PR_2_ increased the expression of iNOS and COX-2, and the expression of pro-inflammatory cytokines such as IL-6, TNF-α and IFN-γ, and chemokines CCL3/MIP-1α, CCL4/MIP-1β, CCL5/RANTES and CXCL10/IP-10 [363]. This shows an immunosuppressive effect of S1P on these cells. However, in mouse bone marrow-derived macrophages, knockout of Gα_12/13_ caused an increase in the expression of iNOS and IL-6 but not COX-2 nor the cytokines IL-1β, IL-10 and TNF-α [363]. At physiological concentrations, S1P alone did not result in an increased production of TNF-α, IL-6, IL-10, IL-12, or CCL2/MCP-1 in bone marrow-derived macrophages or in these cells differentiated to M1 or M2 macrophages [364]. S1P also did not affect phagocytic capacity or iNOS expression in that model.

### FTY720 as an immunosuppressive drug

Knowledge of the effect of S1P on individual immune cells, including cells in the tumor niche, is incomplete. So far, some studies have been based on the influence of FTY720, a compound with complex effects on S1PR. Therefore, here we will discuss the mechanisms of FTY720 action to better understand the effect of S1P on various immune system cells.

2-amino-2-[2-(4-octylphenyl)]-1,3-propanediol hydrochloride (FTY720/fingolimod) is an S1P analogue which exhibits immunosuppressive activity. FTY720 is phosphorylated by both SphK isoforms [365, 366], but phosphorylation by SphK2 has better reaction parameters than SphK1. Therefore, in the human body FTY720 is phosphorylated mainly by SphK2 [367–371]. A reverse reaction, i.e. the dephosphorylation of FTY720-P, is carried out by lipid phosphatase 3 (LPP3) and to a lesser extent by SPP1 in cells [372]. Due to the uneven distribution of both SphK isoforms across human organs, FTY720 is mostly phosphorylated in the spleen, brain and lung [365, 373]. To a lesser degree, phosphorylation occurs in blood and lymph nodes, and is very low in other organs.

FTY720-P is an agonist of S1PR_1_, S1PR_3_, S1PR_4_ and S1PR_5_ [373]. However, studies on S1PR_2_ show that FTY720-P at a concentration of 40nM can activate some signaling pathways through this receptor [374, 375]. Binding affinities of FTY720-P for receptors S1PR_1_, S1PR_3_ and S1PR_5_ are about 10nM, while100nM for S1PR_4_ [373]. At higher concentrations also non-phosphorylated form of FTY720 can activate S1PR_1_ (binding affinities of 300±51nM), and S1PR_5_ (binding affinities 2623 ± 317nM) [376]. In this way, it activates these receptors and acts similarly to S1P. Nanomolar concentrations of FTY720 cause permanent internalization, downregulation and finally degradation of S1PR_1_ and S1PR_5_, and to a lesser extent S1PR_2_ [377]. FTY720-P also shows similar properties against S1PR_1_ [378–380]. As a result, FTY720 and FTY720-P disrupt the signal transmission from these receptors. Eventually, FTY720 is inactivated in the liver via ω-hydroxylation catalyzed by CYP4F2 and to a lesser extent by CYP4F3B [381].

Due to its properties, FTY720 has been investigated as an immunosuppressive agent in organ and tissue transplants [382–386]. In particular, FTY720 accumulates in lymph nodes and inhibits the egress of lymphocytes [387]. This reduces the number of these cells in the blood and thereby reduces the immune response [388]. FTY720 is a potential anti-inflammatory drug in ischemia-reperfusion injury [389, 390]. FTY720 can also penetrate the blood-brain barrier [391], reducing inflammation in the brain [392]. Therefore, it can be used as a drug against relapsing-remitting multiple sclerosis, and has already been approved by the FDA for universal use [393–395].

### Sphingosine-1-phosphate and microglial cells

S1P is a hormone involved in the activation of the microglia by pro-inflammatory factors. An activation of these cells by a pro-inflammatory factor such as LPS results in an increase in the expression and activity of SphK1 and thus an increase in the production of S1P [396]. The effect of LPS on IL-1β and TNF-α production in BV2 microglial cells is cancelled by SphK1 gene knockout, or the use of an inhibitor of this enzyme [397, 398]. Without pro-inflammatory factor LPS, S1P alone only slightly increases TNF-α and IL-1β production [396].

SphK1 gene knockout, or the use of an inhibitor of this enzyme, only partially suppress the effect of LPS on iNOS expression [396]. This shows that SphK1 activity only partially participates in the LPS-induced expression of this enzyme. In contrast, SphK1 gene knockout significantly lowers iNOS expression. Blocking of S1PR_1_ activity does not completely suppress the effect of LPS on the expression of proinflammatory cytokines in microglial cells. Probably, other S1PRs are involved in this mechanism, or, in part, this effect depends on the S1P intracellular pool. However, further research is necessary in this area.

Based on current knowledge, it can be concluded that S1P plays the role of a second messenger within the microglial cell. TNF-α and cerebral ischemia reperfusion and oxygen-glucose deprivation reperfusion result in increased SphK1 activity. As a result, intracellular S1P levels increase, which in turn increases the activity of TNF receptor-associated factor 2 (TRAF2) [345, 346, 347]. The subsequent activation of E3 ubiquitin ligase eventually results in the activation of NF-κB, followed by the expression of genes dependent on this transcription factor. An example of the effect of this signal transmission pathway is the production of IL-17 which acts neurotoxically [347, 399].

The importance of SphK1, S1P and S1PR_1_ for inflammatory reactions in the brain allows for the development of a therapeutic approach that could protect this organ from damage. An example of such a therapeutic approach is the use of FTY720, which has been shown to inhibit LPS-induced microglia activation *in vitro* [397, 400, 401] and *in vivo* in mice with ischemic lesion [402]. FTY720 acts partly via the disruption of S1PR_1_, as demonstrated by the use of W146, an antagonist of this receptor [400]. Due to the effect on S1PRs, FTY720 interferes with signal transmission from p38 MAPK without affecting JNK1/2 MAPK [401].

In addition to the effects on inflammatory cytokines in inflammations, FTY720 increases expression of brain-derived neurotrophic factor (BDNF) and glial cell-derived neurotrophic factor (GDNF), which both have a neuroprotective effect [400]. In contrast, FTY720 does not alter the production of IL-6, IL-10, IL-12p40 and TNF-α in microglial cells activated by CD40L or toll-like receptor 3 (TLR3) ligand [403]. This shows that the effect of S1PRs and so the action of FTY720 can occur only in some immune responses.

### Sphingosine-1-phosphate and myeloid-derived suppressor cells

Myeloid-derived suppressor cells (MDSC) are a very important element in GBM mechanisms. These cells are present in significant numbers in the GBM tumor. It is estimated that they represent 40%±20% of all CD11b^+^ cells in this tumor [176]. GBM patients also have an elevated number of these cells in the blood. MDSC are mainly involved in cancer immune evasion but also in angiogenesis and cancer cell migration [404, 405].

In the functioning of MDSC, an important role is played by S1P, as evidenced by experiments involving FTY720. This drug caused an *in vivo* increase in MDSC activity in the spleen of murine sclerodermatous chronic graft-versus-host disease [406] and in the spleen and liver of the immune-mediated hepatic injury model [407] and in tumors [408]. Due to these properties, FTY720 silences the immune response, protecting the organs from damage, but also participating in tumor processes.

MDSC accumulation is dependent on increased expression of CXCL1/GROα and CXCL2/GROβ as well as increased expression on the MDSC receptor for these chemokines: CXCR2 [407]. In the tumor, activation of the S1PR_3_-ERK1/2 MAPK pathway on MDSC by FTY720 results in increased expression of granulocyte-macrophage colony-stimulating factor (GM-CSF), resulting in MDSC accumulation in the tumor niche and autocrine stimulation of immunosuppressive functions of these cells [408–410]. These results show that S1PR_3_ activation by carcinogenic S1P can stimulate MDSC immunosuppressive activity in the tumor niche.

Prolonged exposure to FTY720 also causes a disturbance in the transmission of signals from S1PR_1_ that independently of PI3K-PKB reduces the activity of mTOR, thus increasing the expression of iNOS in the MDSC [407]. An increase in the concentration of NO causes the differentiation and stimulation of T_reg_ function, which is as an important mechanism of the immunosuppressive effect of FTY720 on these cells. However, such an action can help facilitate the development of cancer [411, 412].

The balance of the effect of S1P on S1PR_1_ and S1PR_3_ in the activity of MDSC in tumor niche requires further studies.

### Sphingosine-1-phosphate and regulatory T cells

S1P also influences the function of T_reg_ cells. In naïve CD4^+^ T cells S1PR_1_ causes activation of mTOR [413]. The activation of this pathway causes Smad3 malfunction. Thus, S1P and FTY720 disrupt *in vitro* and *in vivo* cell differentiation to T_reg_ but stimulate differentiation into cytotoxic T_H_1. This mechanism is an element of a negative feedback mechanism that inhibits overly extensive TGF-β action, as the latter causes an increase in SphK1 expression. S1P then disrupts the action of TGF-β [413]. However, FTY720 in other experimental conditions causes permanent down-regulation and degradation of S1PR_1_ which interferes with signal transduction through the receptor [377–380]. This enhances the immunosuppressive effect.

*In vivo* experiments show that FTY720 causes differentiation and an increase in T_reg_ numbers in the spleen [414], but FTY720 does not affect the proliferation of these cells [414, 415]. Wolf et al. have shown that FTY720 does disrupt T_reg_ proliferation by inhibiting IL-2-dependent STAT5 phosphorylation [416]. In inflammatory reactions, FTY720 causes *in vivo* retention of T_reg_ in lymph nodes near the inflammatory sites, but not from the spleen [417]. An increase in T_reg_ numbers results in an immune response near the lymph nodes [418].

S1P participates in the silencing of the immune response in the tumor microenvironment. S1P causes the S1PR_1_-mediated activation of STAT3, and thereby accumulation of T_reg_ in the tumor niche, as evidenced in an *in vivo* model of the B16 melanoma cell line, MB49 bladder carcinoma line, and in patients with breast cancer [419, 420]. So far no study has analyzed the effect of S1P on T_reg_ in the tumor niche. Research on FTY720 show that this drug supports the functions of these cells. The active form of FTY720-P induces an increased expression of TGF-β1 and FoxP3 marker in T_reg_ cells [415, 421]. On the other hand it does not cause a significant increase in IL-10 production in these cells. In addition, FTY720 expresses the Foxp3 marker on Foxp3^−^CD4^+^T cells *in vivo* [417]. This indicates that S1P has an opposite, anti-cancer effect by disturbing the functions of T_reg_.

### Sphingosine-1-phosphate and neutrophils

The effect of S1P on neutrophils in the tumor microenvironment is poorly understood. Also, the effect of S1P on neutrophil functions is still debatable. *In vivo* and *in vitro* murine models showed that the knockout of SphK1 or SphK2 had no effect on the migration and respiratory burst of neutrophils [422]. However, other experiments show the importance of the SphK1-S1P pathway in the physiology of these cells. The activation of S1PR_1_ results in the *in vivo* infiltration of neutrophils during inflammatory reactions [423]. Different inflammatory reactions depend on different mechanisms. During an allergic response, neutrophil infiltration is dependent on SphK1 activity but not on S1PR_1_, S1PR_2_ and S1PR_3_ activity [424].

The direct effect of S1P on neutrophils consists of a moderate inhibition of neutrophil migration via HUVEC and inhibits chemotaxis stimulated by IL-8/CXCL8 or formyl-methionyl-leucyl-phenylalanine (fMLP) [425]. Indirectly, S1P acts on neutrophil migration by causing increased expression of IL-8/CXCL8, a chemokine acting on neutrophils. The effect of S1P on IL-8/CXCL8 production has been demonstrated in normal epithelial virus-transformed BEAS-2B cell line [426–428], A549 lung carcinoma line [429] and human airway smooth muscle [430]. This mechanism is involved in airway inflammation. In BEAS-2B cells the effect of S1P on the expression of IL-8/CXCL8 depends on the activation S1PR_2_ [428]. This enables the activation of NF-κB and an increase in IL-8/CXCL8 expression. Importantly, this effect is independent of EGFR. In BEAS-2B cells S1P can also activate ERK1/2 MAPK, depending on phospholipase D (PLD) in these cells [426, 427]. ERK1/2 MAPK and PLD activation may also involve an increase in intracellular Ca^2+^ concentration, as demonstrated in experiments on A549 cells [429].

Activation of ERK1/2 MAPK results in an increase in IL-8/CXCL8 expression. The mechanism of S1P effect on IL-8/CXCL8 expression is cell dependent. In HUVEC S1P increases expression of IL-8/CXCL8 by activating S1PR_1_ and S1PR_3_ [343]. In human airway smooth muscle isolated from patients, the effect of S1P on the expression of IL-8/CXCL8 was dependent on p38 and ERK1/2 MAPK, but independent of NF-κB [430]. p38 and ERK1/2 MAPK activate mitogen and stress activated kinase 1 (MSK1) which results in an increase in IL-8/CXCL8 expression.

In addition to the effects on chemokines, S1P increases the expression of ICAM-1 on cells such as A549 [429] and HUVECs [343]. This helps in the diapedesis of neutrophils. S1P also increases IL-8/CXCL8 expression in ovarian cancer cells such as HEY, OCC1 and SKOV3 [431]. The effect of S1P on cells in the GBM niche requires further studies. S1P is mainly synthesized by GSC [305]. If S1P exerts a chemotactic effect on neutrophils via IL-8/CXCL8 then this may explain the presence of these cells near the GSC [185]. However, the association of S1P with the recruitment and distribution of neutrophils in GBM has yet to be investigated.

Neutrophils are short-lived cells that undergo rapid apoptosis [432]. Activation of these cells by pro-inflammatory factors blocks the apoptosis, with an important role played by SphK1: an LPS-induced increase in the expression and activity of SphK1 inhibits the intensity of neutrophil apoptosis via activation of PI3K [433] and p38 MAPK [434, 435]. A similar mechanism occurs in the activity of GM-CSF [433].

Extracellular S1P and SphK1 activity in cells increases the respiratory burst in activated neutrophils. In particular, studies on neutrophil activation by fMLP [433, 436, 437] and activation of the receptor for immunoglobulin Fcγ [438] show an increase in the production of S1P in immune responses, which augments the respiratory burst in activated neutrophils. S1P affects the activity of NADPH oxidase in two ways. It activates the PI3K-PKB pathway [433, 437] and independently of PI3K it increases intracellular Ca^2+^ concentration [433, 434]. The increase in intracellular Ca^2+^ concentration results in activation of p38 MAPK and consequently S100A8/A9 translocation and thereby an increase in NADPH oxidase activity [434]. However, this impact still requires further research because Zemann et al. had earlier shown that a knockout of the SphK1 gene did not affect the intensity of the respiratory burst induced by fMLP [422].

Enzymes involved in S1P production may also inhibit the respiratory burst. In particular, LPS causes increased expression of SphK1 in neutrophils [439]. This protein, regardless of its enzymatic activity, stabilizes JNK MAPK and thus distorts the signal transmission through this kinase. Consequently, it reduces NADPH oxidase activation.

Neutrophils accumulate in GBM tumors, which results in a deterioration in prognosis for patients [186, 187]. In the tumor niche, neutrophils secrete many substances involved in angiogenesis, migration and invasion of tumor cells and in tumor immune evasion [127, 440]. Nevertheless, the significance of these cells in the context of cancer processes is poorly understood. The impact of S1P on tumor neutrophils is even less understood. However, *in vitro* studies show that S1P activity on neutrophil is similar to the behavior (migration and apoptosis inhibition) of these cells in the tumor niche. Significantly, the respiratory burst in neutrophils associated with cancers is at a low level [127]. S1P does increase the respiratory burst, but in the tumor microenvironment there are no substances that stimulate it.

## MULTI-DRUG THERAPY AGAINST SECRETORY FACTORS

### Therapeutic strategies for the treatment of glioblastoma multiforme

Treatment limitations such as high average age onset, tumor localization, and still inadequate knowledge of GBM pathophysiology, are cited as factors contributing to the short median survival [441]. Currently, standard therapeutic procedures in GBM include surgical resection of tumors followed by radiotherapy and chemotherapy. Surveys so far confirm that tumor resection should be performed to the maximum extent possible [442]. The next step in GBM treatment is radiotherapy, i.e. external beam radiation therapy [443] or stereotactic radiosurgery (gamma knife) [444]. Radiotherapy is combined with chemotherapy, in particular fotemustine or cyclically administered TMZ [445, 446]. Both these compounds are alkylating agents and thus, by damaging DNA, they inhibit cell proliferation. Nevertheless, the currently used therapeutic approach to GBM treatment is very ineffective, with very low 5-year survival [4]. Therefore, new therapeutic methods are being sought.

### Novel therapies

Novel therapies are being developed to support the classic GBM treatment. Many of these therapies are still at clinical level [447]. The novel therapies include, among others, calorie restricted ketogenic diet [448–451], immunotherapy [452–455] and the use of oncolytic viruses [456–458]. New chemotherapeutics are also being developed to generate personalized therapy [459–460].

### Calorie restricted ketogenic diet

Changes in the metabolism of carbohydrates and fats are one of the ‘hallmarks of cancer’ [13, 14]. First demonstrated by Otto Warburg, after whom it was named the Warburg effect [461, 462], the phenomenon is based on the intense anaerobic glycolysis that produces lactic acid and acidification in the tumor microenvironment. Lactic acid and low pH in the tumor are one of the most important elements of the tumor microenvironment which cause cancer immune evasion [463]. To some extent, the Warburg effect also makes tumor cells dependent on carbohydrates as a major source of energy, as cancer cells are not able to use ketone bodies as a source of energy. Therefore the implementation of the ketogenic diet, i.e. carbohydrate-restricted diet, causes the ‘starvation’ of cancer cells, including GBM [449, 464]. Normal cells, including nerve cells, are able to metabolize ketone bodies. Due to the metabolic difference between GBM and non-cancer cells, a combination of a calorie restricted ketogenic diet with a standard therapeutic approach is proposed [448–451].

### Immunotherapy

Certain hopes are also linked to two therapeutic approaches, which may act on non-cancer cells or directly on tumor cells. The first approach targets cells associated with the tumor, particularly T_reg_, macrophages and microglia, which have a significant effect on tumor immune evasion [59, 465]. The second approach aims at stimulating the cells of the immune system to destroy cancer cells [452–455]. The combination of these two strategies is also advocated because of tumor immune evasion processes that compromise the effects of immunotherapy [238, 454, 466]. Therefore, the use of antitumor immunostimulant drugs, especially the use of pro-inflammatory cytokines, should increase the therapeutic effects of immunotherapy. This therapeutic approach, as well as immunotherapy itself, specifically destroy tumor cells. As a result, it has fewer side effects compared to non-specific drugs destroying dividing cells [467–470].

### Multi-drug therapy as a strategy against glioblastoma multiforme: personalized therapy

The ongoing research on GBM continues to reveal specific mechanisms in the development of GBM, which helps develop therapies targeted at a specific enzyme, tissue hormone, or other specific tumorigenic agent in a particular patient. This is known as personalized therapy [459, 460].

Nevertheless, GBM is a tumor with a very high intratumoral heterogeneity. GBM cells in each patient exhibit a different sensitivity to a given drug. It is estimated that 1/4 of all GBM cells in a given patient are resistant to TMZ and 1/10 are very susceptible to this drug [22]. Therefore, the use of a single drug in GBM results in unsatisfactory therapeutic outcomes. An example of this is TMZ, which, when given to patients undergoing radiotherapy and neurosurgical intervention, results in an increase in the 5-year survival from 1.9% to 9.8% [4]. One also should not forget about the serious side effect of antineoplastic drugs. The use of many drugs and therapeutic approaches at the same time will result in compounding side effects [471].

### Multi-drug therapy as a strategy against intratumoral heterogeneity

The extension of the personalized therapy may be a multi-drug therapy, with particular emphasis on the secretory factors in a tumor. Using only one drug often causes GBM recurrence, because a significant percentage of tumor cells are resistant to the drug [22]. It is much less likely to find a tumor cell resistant to two drugs at the same time, and even less so to five drugs. If TMZ is used in addition to radiotherapy, it can increase the 5-year survival rate 5 times. The use of an additional drug can further increase this rate [308, 472–476]. It is best to include a drug that attacks a GBM specific target that does not have a significant function in healthy cells. As a result, the side effects of this drug will be smaller. One example of this is the use of drugs against CMV infection [475, 477, 478].

When choosing drugs for a multidrug therapy, how they interwork should be considered. One should be chosen from the ‘hallmarks of cancer’ and then match all drugs to the selected target. At the same time, GBM contains many mechanisms that trigger the stimulation of proliferation, apoptosis inhibition or tumor immune evasion. The use of four drugs inhibiting proliferation and one specifically impairing tumor immune evasion results in the response of tumor cells similar to when only anti-proliferative drugs are used. Tumor immune evasion mechanisms vary in the tumor microenvironment. Blocking of one signaling particle leads to the drug's action only in a small part of the tumor (i.e. due to intratumoral heterogeneity), or a lack of therapeutic effects associated with the complementary action of other immune evasion mechanisms.

NT, S1P, GDF-15 and CMV infection have almost identical properties and functions. Within GBM, their concentrations are increased, and the expression of their receptors and enzyme activity responsible for their production also increase. All these factors have implications for all significant ‘hallmarks of cancer’ such as stimulated proliferation, inhibited apoptosis, tumorigenic effect on GSC, angiogenesis, migration, invasion, and tumor immune evasion. In addition, the increase in the concentrations of these factors is not local, but gradually occurs throughout the tumor. This offsets certain problems associated with intratumoral heterogeneity. One may even assume that in the tumor microenvironment there is a pool of all the secretory factors that complement and cooperate with one another. Therefore, multi-drug therapy may be used to interfere with various secretory factors. As a result, tumorigenic and antitumoral imbalance in the tumor microenvironment may be impaired, consequently leading to the destruction of all tumor cells [479].

### Antineoplastic agents fighting cytomegalovirus infection

Based on knowledge used to develop the currently used therapies, cytostatics are used to treat cancer [480]. These drugs or X-rays destroy only dividing cells. Due to the fact that GSC are rarely-dividing cells with drug resistance enzymes, this therapeutic approach has only the short-term effect of decreasing tumor mass [481]. During such therapy, GSC are not destroyed, which results in the recurrence of cancer. Evaluated on the basis of available literature, the role of CMV in tumoral mechanisms in GBM brings some therapeutic hopes. In particular, tropism of CMV for CD133^+^ GSC and the significance of this virus in GSC functions make these cells significant in CMV/GBM therapy [46, 48–50].

The growth of a tumor associated with chronic CMV infection takes years. During this process Darwinian-like selection of cells occurs, in terms of tumor processes, resulting in the formation of advanced cancer [12]. In these, tumor processes are fully dependent on the pro-tumor properties of CMV. This leads to the susceptibility of such tumors to antiviral drugs used against CMV [473, 475]. Currently, the proposed approach is to combine radiotherapy and TMZ with the use of antiviral drugs or immunotherapy against CMV.

Cidofovir and valganciclovir are being tested as antiviral drugs in CMV infection, while other new drugs are also being developed. Cidofovir is an analog of cytosine. It inhibits DNA polymerase activity not only in CMV but also in other viruses [482]. This counteracts CMV replication. However, the activity of cidofovir is very non-specific [473]. This drug is also a substrate for non-viral DNA polymerases in dividing cells. As a result, cidofovir causes *in vitro* DNA double-stranded breaks and apoptosis of U-87 MG and SF7796 cells, independently of CMV infection [473]. Also, this drug *in vivo* enhances the survival of athymic mice intracranially inoculated with U-87 MG and SF7796 cells [473].

Another anti-CMV drug tested against GBM is valganciclovir. This drug is specifically phosphorylated by the UL97 kinase viral protein [482]. This reaction is necessary to convert this prodrug into active ganciclovir. Because valganciclovir penetrates the blood-brain barrier, it can be used in GBM therapy [483, 484]. Combined with standard therapy in clinical trials, valganciclovir brings a significant increase in mean survival rate. The effects of valganciclovir can occur after only 6 months of therapy with this prodrug. At this point the 4 year postoperative survival and median overall survival increase from 5.9% and 13.1 months to 27.3% and 24.1 months, respectively [475]. Continuation of valganciclovir therapy can significantly increase median overall survival to 56.4 months [474]. Also the combination of valganciclovir with bevacizumab, radiotherapy and TMZ increases the 6-month progression-free survival and average survival [485].

In addition to the use of antiviral drugs, researchers also recommend the use of immunotherapy against CMV in GBM treatment, especially the use of autologous dendritic cells [466] or autologous cytotoxic T cells [478, 486–488] vaccinated with specific CMV antigens. Autologous dendritic cells are sensitized to the pp65 viral protein and then are introduced into the body of the patient. The combination of this therapeutic approach with neurosurgery, radiotherapy and TMZ increases overall survival from 19.2 months to 41.1 months and long-term progression-free survival from 8.0 months to 25.3 months [466]. In addition to dendritic cells, CMV/GBM immunotherapy uses autologous cytotoxic T cells that are sensitized to CMV antigens by autologous dendritic cells [488–490] or autologous peripheral blood mononuclear cells [478, 486, 487]. The use of autologous cytotoxic T cells in GBM therapy increases the mean overall survival from 4.3 months to 79.8 months [478].

### Antitumor agents directed against neurotensin and neurotensin receptor

Many anti-cancer drugs directed against NT and NTSR_1_ (Table 2) are currently being tested. In the research of new GBM therapies, the most significant in this group of compounds is a NTSR_1_ antagonist: SR48692 [202, 491–493]. This compound exhibits antitumor properties *in vitro* by inhibiting proliferation and cell migration of U-87 MG GBM cells and GL261 gliomas [202]. Also SR48692 has therapeutic properties *in vivo* in C57BL/6 mice intracranially inoculated with GL261 cells [202].

**Table 2:** Experimental anti-cancer drugs and pharmaceutical agents against NT and NTSR_1_

Drug	Mechanism of action	Research model	Bibliography
SR48692	NTSR_1_ antagonist	A375 melanoma cell line *in vitro* **GL261 murine glioma cell line *in vitro* and *in vivo*** NCI-H209 small cell lung cancer cells *in vitro* and *in vivo* PANC-1 pancreatic cell line *in vitro* **U-87 MG glioblastoma cell line *in vitro***	202, 491-493
Neurotensin analogs	NTR_1_-targeted drug	HT-29 colorectal adenocarcinoma cell line *in vitro* and *in vivo* NCI-H446 small cell lung cancer cells *in vitro* and *in vivo* WiDr colorectal adenocarcinoma cell line *in vitro* and *in vivo* Ductal pancreatic adenocarcinoma clinical trial	494-499
DOTA- and DTPA- chelated neurotensin analogs	NTR_1_-targeted drug	HT-29 colorectal adenocarcinoma cell line *in vitro* and *in vivo*	500-502
Neurotensin Branched Peptides	NTR_1_-targeted drug	HT-29 human adenocarcinoma cell line *in vitro* and *in vivo* HT-1376 bladder cancer cell line *in vitro* and *in vivo*	503, 504
Neurotensin polyplex	Gene transfection	N1E-115 neuroblastoma cell line *in vitro* and *in vivo*	505-507

Compounds that destroy tumor cells which overexpress NTSRs are also being tested on models of other tumors. These are NT derivatives labelled with radioactive isotopes or cytostatic drugs such as methotrexate or gemcitabine. An example of NT derivatives is a modified fragment of this hormone that does not undergo rapid proteolytic degradation [494–499]. Such an NT analog may be further chelated by diethylenetriamine pentaacetic acid (DTPA) or 1,4,7,10-tetraazacyldodecane-1,4,7,10-tetraacetic acid (DOTA) to enhance stability [500–502]. Another possibility are oligobranched peptides containing a NT fragment in their sequence which is recognized by NTSR_1_ [503, 504]. By labeling such NT derivatives with radioactive isotopes or cytostatic drugs, such drugs specifically destroy tumor cells that overexpress NTSR_1_. In addition to this therapeutic approach, a gene therapy is being tested in which a NT polyplex is used, i.e. a vector composed of NT, poly-L-lysine, and a plasmid encoding an antitumor protein such as thymidine kinase [505–507]. Nevertheless, these NT derivatives, used in the treatment of other cancers, do not cross the blood-brain barrier and so cannot be used in GBM therapy. Hence the search for the new methods of weakening the blood-brain barrier or carrying drugs through this barrier [508–511].

### Drugs directed against growth differentiation factor-15

The GDF-15 receptor is currently unknown. Therefore, the most important route in anticancer therapy directed against this secretory factor are antibodies neutralizing GDF-15 [512]. Nevertheless, the blood-brain barrier prevents the use of these antibodies in GBM therapy [509–511].

### Drugs targeted at the sphingosine-1-phosphate pathway

In anti-cancer therapy directed against S1P, much attention is given to the inhibitors of SphKs [513–516]. In particular, the best known is the specific inhibitor SphK1: 2R,3S,4E)-N-methyl-5-(4’-pentylphenyl)-2-aminopent-4-ene-1,3-diol (SK1-I) both SphK: 2-(p-hydroxyanilino)-4-p-chlorophenyl)thiazole (SKI-II) [517, 518]. Their efficacy against GBM has also been confirmed *in vitro* on various cell lines such as A-172, LN-18, LN-229, U-87 MG, U-251 MG and T98G [301, 302, 308, 472]. Also on the *in vivo* model, SphKs inhibitors have shown antitumor properties against GBM. SK1-I reduces tumor mass, inhibits angiogenesis and causes apoptosis of tumor cells, and increases the survival of nude mice intracranially inoculated with LN-229 cells [301]. In addition, SKI-II destroys tumor cells *in vivo* in nude mice inoculated subcutaneously with U-87 MG cells [302]. In GBM therapy with SphK inhibitors, it is also proposed to combine these drugs with the currently applied therapy, in particular with TMZ, to increase the therapeutic effect [308, 472].

*In vitro* and *in vivo* studies indicate that FTY720/fingolimod micromolar concentration has antitumor properties [519, 520]. FTY720 *in vitro* inhibits proliferation and migration, and causes apoptosis of GBM cell lines such as U-87 MG, U-251 MG, T98G and GSC isolated from GBM tumors [521–525]. This *in vivo* compound reduces tumor mass, causes apoptosis and necrosis of tumor cells, and increases survival of nude mice intracranially inoculated with GSC from GBM tumors [522]. Also, FTY720 produces the same effects in nude mice subcutaneously inoculated with U-87 MG and U-251 MG cells [524]. FTY720 penetrates the blood-brain barrier and can therefore be used in GBM therapy [391, 522, 524]. Activation of S1PRs causes GBM cell proliferation. Nevertheless, tumor cells are characterized by frequent mutations in the p53 protein [526]. This results in a lack of stimulation of cell proliferation by S1PRs activation [304] and thereby enhances the antitumor activity of FTY720 that is dependent and independent of these receptors [520]. At nanomolar concentrations, FTY720 causes down-regulation and degradation of S1PRs [377]. Its antineoplastic properties can only be observed at micromolar concentrations, which indicates the the mechanisms of the antineoplastic action of FTY720 is independent of S1RPs

FTY720 also inhibits angiogenesis and cancels the action of VEGF by reducing vascular permeability and reduced sprouting of HUVEC at concentrations below 1 nM, by acting on CXCR4 and S1PR [367, 527]. CXCR4 receptors are receptors whose activation may be involved in angiogenesis. This receptor may be regulated by S1PRs [528–530]. Nevertheless, the effect of FTY720 on CXCR4 in angiogenesis inhibition should be further explored. Also in an *in vivo* model, FTY720 inhibited tumor growth and angiogenesis in mice inoculated with PLC/PRF/5 and Huh7 human hepatocellular carcinoma lines at a dose of 10mg/kg per day [531], B16/BL6 murine melanoma at 3mg/kg daily [378], and Lewis lung carcinoma LLC1 line at a dose of 10 mg/kg daily [527].

However, the GBM tumor does not consist only of tumor cells but also of tumor-associated cells, in particular immune cells. Immune reactions also play a very important role in tackling cancer. The use of immunosuppressive drugs such as FTY720 results in the impairment of the immune system and consequently may facilitate the development of GBM as well as other tumors [411, 412].

In addition to SphK inhibitors, researchers postulate the use of S1PR antagonists in the treatment of tumors [532, 533]. It is also advocated to use S1P-neutralizing antibodies acting on many types of cancer [534–537]. However, this therapeutic approach has not been studied in terms of glioma and GBM, because the blood-brain barrier significantly impedes the transmission of antibodies to the microenvironment of these tumors. On the other hand, some hope may lie in the search for new methods of transmitting various substances through the barrier [509–511].

### Anti-cancer drugs directed against other secretory factors

Nonsteroidal anti-inflammatory drugs (NSAIDs), in particular selective COX-2 inhibitors and nonselective cyclooxygenase inhibitors, have been reported to reduce the production of PGE_2_ [359, 360, 538–540], or CD39 and CD73 inhibitors reducing adenosine production [541–544]. All of the therapeutic agents that have been mentioned so far can be used in combination with drugs that interfere with the action of tissue hormones with a greater importance for GBM, for example, tyrosine kinase inhibitors or anti-EGFR or anti-EGFRvIII antibodies [545–547].

#### Problem I: blood-brain barrier

The blood-brain barrier protects the central nervous system against various toxic and biological chemicals. It is impervious to antibodies and a significant number of drugs [509–511]. This greatly hampers the treatment of diseases in this organ. Although within GBM the barrier is suppressed, some GBM parts are still protected by it [509–511]. Therefore, NT labelled with radioactive isotopes or cytostatic drugs, as well as specific anti-S1P or anti-GDF-15 antibodies or some of the aforementioned anti-cancer drugs, are ineffective in GBM therapy [507]. This is why researchers are looking for new therapeutic substances that are able to penetrate this barrier. Another field is the search for drugs that would weaken the action of the blood-brain barrier, or for compounds that would carry conjugated substances through this barrier. One example is angiopep-2, which carries NT through this barrier [508]. Advancement of knowledge in solving this problem is necessary in the development of new therapeutic methods in GBM.

#### Problem II: compounding side effects

The use of multidrug therapies that target physiological factors presents a high risk of side effects compounding [471]. Therefore, lower concentrations of all drugs should be used so that only fewer of the enzymes are inactivated. Lower antibody concentrations should also neutralize some of the hormones. As a result of the development of tumoral processes, there are many more aforementioned enzymes, tissue hormones or receptors in the tumor niche or a cancer cell then in non-cancer tissue [202, 262, 263, 288, 289, 292–294]. If these molecules are chosen as the target of therapy, it is more likely that the drug acts at lower concentrations in the tumor cell than in a healthy cell [493]. In this case, the effect of such a therapy on cancer cells would be more toxic than for healthy cells. By reducing the concentration of secretory factors such as NT, S1P, GDF-15, from very high to physiological or even lower levels, it may have a destructive effect on the viability of the tumor cell. In contrast, in the healthy cell a slight decrease in the aforementioned hormones is going to have a much smaller adverse effect. The tumor microenvironment selects tumor cells in a certain direction [479]. As a result, tumor cells are dependent on this environment, in particular on secretory factors, which are very often elevated during cancer development and act on all ‘hallmarks of cancer’.

#### Problem III: therapy duration

GBM is a cancer that recurs despite surgical intervention, radiotherapy and chemotherapy. This is associated with the dissemination of cancer cells across many areas of the brain. After the excision of the main tumor, tumor cells are distributed throughout the entire brain. Over time, they become activated and a new relapse site emerges. Therefore, the effects of some experimental therapies are only visible after more than 6 months of taking the drug [474, 475].

A therapeutic approach based on the interference with the tumor microenvironment disturbs the development of cancer and the formation of a relapse site. Nevertheless, to achieve some therapeutic success, it is required to destroy all cancer cells which create relapse sites. Therefore, therapy must last until this goal is completed.

## CONCLUSION - INTRATUMORAL HETEROGENEITY AS A TARGET OF RESEARCH

To better understand the effects of multidrug therapies, it is important to focus on the changes that occur in tumor processes, and in particular those that lead to intratumoral heterogeneity. Many changes in cancer cells are interrelated, and so GBM tumor cell subtypes exhibit specialization and play different functions [20, 21]. The discovery of patterns of changes in tumor cells will help divide them according to their susceptibility to particular drugs [548]. This will also allow an understanding of the interrelationships between the individual cells in the tumor and, consequently an ability to interfere with the communication between the cells [20, 21]. In this way, it will be possible to develop adequate multidrug therapies with 100% effectiveness and minimal side effects.

## Abbreviations

ATF5: Activating transcription factor 5
CCL: CC motif chemokine ligand
CDK: Cyclin-dependent kinase
CMV: Cytomegalovirus
CTGF: Connective tissue growth factor
CX3CL: C-X3-C motif chemokine ligand
CXCL: C-X-C motif chemokine ligand
EGF: Epidermal growth factor
EGFR: Epidermal growth factor receptor
ERK1/2: Extracellular signal-regulated kinase 1 and 2
FAK: Focal adhesion kinase
GBM: Glioblastoma multiforme
GDF-15: Growth differentiation factor-15
GSC: Glioblastoma stem cells
HIF: Hypoxia inducible factor
HLA: Human leukocyte antigen
HO-1: Heme oxygenase 1
HUVEC: Human umbilical vein endothelial cells
IDO: Indoleamine 2,3-dioxygenase
IE86: Immediate early 86
IGF-1R: Insulin-like growth factor 1 receptor
IL: Interleukin
LPS: Lipopolysaccharide
MAPK: Mitogen-activated protein kinase
MCP-1: Monocyte chemoattractant protein 1
MHC: Major histocompatibility complex
MIP: Macrophage inflammatory protein
MMP: Matrix metalloproteinase
NT: Neurotensin
NTSR_1-4_: NT receptor subtype 1-4
PAI-1: Plasminogen activator inhibitor-1
PDGF: Platelet-derived growth factor
PI3K: Phosphatidylinositol-4,5-bisphosphate 3-kinase
PKB: Protein kinase B
PKC: Protein kinase C
PLC-β: Phospholipase C-β
PPARγ: Peroxisome proliferator-activated receptor γ
RANTES: Regulated on activation,normal T-cell expressed and secreted
S1P: Sphingosine-1-phosphate
S1PR_1-5_: S1P receptor 1-5
sNTSR_3_: Soluble NT receptor subtype 3
SphK: Sphingosine kinase
SPL: S1P lyase
SPP: S1P-catalyzed phosphohydrolase
STAT: Signal transducer and activator of transcription
TAM: Tumor-associated macrophages
TGF-β: Transforming growth factor β
TMZ: Temozolomide
TNF-α: Tumor necrosis factor α
UPA: Urokinase-type plasminogen activator
uPAR: Receptor for urokinase-type plasminogen activator
VEGF: Vascular endothelial growth factor
vIL-10: Viral interleukin-10.
